# A systematic review and meta analysis of measurement properties for the flexion relaxation ratio in people with and without non specific spine pain

**DOI:** 10.1038/s41598-024-52900-z

**Published:** 2024-02-08

**Authors:** Diana De Carvalho, Sarah Mackey, Daphne To, Allyson Summers, Mona Frey, Kristen Romme, Sheilah Hogg-Johnson, Samuel J. Howarth

**Affiliations:** 1 Division of Population Health and Applied Health Sciences, Faculty of Medicine, Memorial University of Newfoundland, St. John’s, NL Canada; 2 Department of Clinical Education, Canadian Memorial Chiropractic College, Toronto, ON Canada; 3 Division of BioMedical Sciences, Faculty of Medicine, Memorial University of Newfoundland, St. John’s, NL Canada; 4 Health Sciences Library, Faculty of Medicine, Memorial University of Newfoundland, St. John’s, NL Canada; 5 Division of Research and Innovation, Canadian Memorial Chiropractic College, Toronto, ON Canada

**Keywords:** Musculoskeletal system, Diagnostic markers

## Abstract

This review sought to identify, critically appraise, compare, and summarize the literature on the reliability, discriminative validity and responsiveness of the flexion relaxation ratio (FRR) in adults (≥ 18 years old) with or without spine pain (any duration), in either a clinical or research context. The review protocol was registered on Open Science Framework (https://doi.org/10.17605/OSF.IO/27EDF) and follows COSMIN, PRISMA, and PRESS guidelines. Six databases were searched from inception to June 1, 2022. The search string was developed by content experts and a health services librarian. Two pairs of reviewers independently completed titles/abstracts and full text screening for inclusion, data extraction, and risk of bias assessment (COSMIN RoB Toolkit). At all stages, discrepancies were resolved through consensus meetings. Data were pooled where possible with a three-level random effects meta-analyses and a modified GRADE assessment was used for the summary of findings. Following duplicate removal, 728 titles/abstracts and 219 full texts were screened with 23 included in this review. We found, with moderate certainty of evidence, that the cervical FRR has high test–retest reliability and lumbar FRR has moderate to high test–retest reliability, and with high certainty of evidence that the cervical and lumbar FRR can discriminate between healthy and clinical groups (standardized mean difference − 1.16 [95% CI − 2.00, − 0.32] and − 1.21 [− 1.84, − 0.58] respectively). There was not enough evidence to summarize findings for thoracic FRR discriminative validity or the standard error of measurement for the FRR. Several studies used FRR assuming responsiveness, but no studies were designed in a way that could confirm responsiveness. The evidence supports adequate reliability of FRR for the cervical and lumbar spine, and discriminative validity for the cervical and lumbar spine only. Improvements in study design and reporting are needed to strengthen the evidence base to determine the remaining measurement properties of this outcome.

## Introduction

Non-specific spine pain defined as neck or low back pain in the absence of a pathological cause is a leading contributor to disability worldwide and affects individuals of all ages^1^. Using “biomarkers” to sub-classify patients with non-specific spine pain has been an avenue of interest to guide clinical management^2^. Two recent systematic reviews have suggested that the flexion-relaxation ratio (FRR), quantified using features of electromyographic (EMG) signals from the lumbar spine’s extensor muscles during full forward bending, to be a potential biomarker of neuromuscular function for people with non-specific chronic low back pain (NSCLBP)^3,4^. Similar features of EMG signals from the cervical extensor muscles have also been used in research studies conducted using people with non-specific neck pain. Viability of the FRR as a biomarker for use in people with non-specific neck or low back pain is predicated by its psychometric properties such as reliability, validity, and responsiveness; however, these parameters have yet to be fully reported.

The typical pattern of spine extensor EMG signals during forward bending is characterized by increased activity to eccentrically control movement of either the head (cervical) or trunk (lumbar) followed by the sudden quiescence of these muscles near the end-range of motion^5^. A second period of activity is observed for the muscles to concentrically extend the head or trunk from the fully flexed posture to an upright posture. Previous work has reported that the reduction in EMG signal magnitude near the end-range of motion is not exhibited by some individuals with non-specific spine pain^6,7^. Using the EMG signal magnitude, typically expressed in units of (milli)volts, at full forward flexion as a biomarker is problematic because of the many factors that can influence EMG signal magnitude^8^. Measures of EMG signal magnitude during either the forward bending or return phases of the movement can be used to provide a reference for the EMG signal magnitude at full forward flexion. The ratio of these measures of EMG signal magnitude defines either the flexion-relaxation ratio (FRR; when the forward bending phase is used as the reference) or extension-relaxation ratio (ERR; when the return phase of the movement is used).

As previously mentioned, two recent systematic reviews have investigated the psychometric properties of the FRR for people with NSCLBP^3,4^. These reviews focused on reliability (inter-rater, intra-rater, test–retest within and between sessions), validity (content, criterion, construct/discriminative) and prevalence of the FRR. Neither responsiveness, which refers to the ability of a measurement to detect change over time when there has been a change (e.g., response to treatment or progression of disease) in the construct being measured^9^ nor standard error of the measure (e.g., the spread of scores around the true score) were evaluated by either of the previous systematic reviews. Furthermore, there has not been a systematic review or meta-analysis to our knowledge that has focused on the psychometric properties of the FRR or ERR for the cervical extensor muscles. Thus, the objective of this review was to identify, critically appraise, compare, and summarize the literature on the construct validity (hypothesis testing for discriminative validity), reliability (test–retest, intra-rater, inter-rater), measurement error, and responsiveness of the FRR in adults (≥ 18 years old) with or without spine pain (any duration), in either a clinical or research context.

## Results

### Study selection

**Figure 1:**
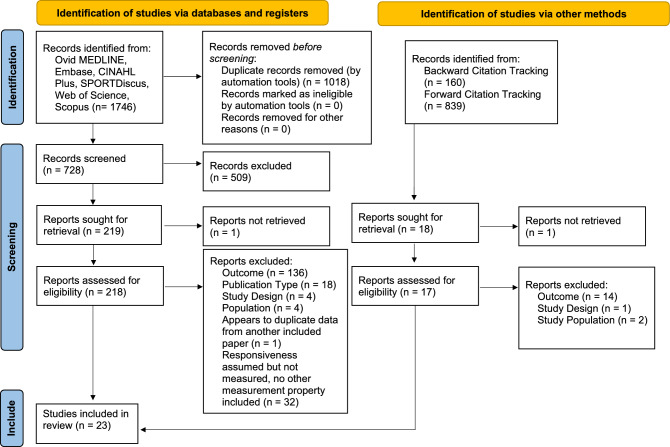
PRISMA flow diagram.

Our search identified 1746 articles. Following duplicate removal, we screened 728 titles/abstracts and 219 full texts. Backward and forward citation tracking identified 999 articles that were screened, leading to 17 additional full texts that were reviewed but found no studies for inclusion. The full text for two studies could not be retrieved^10,11^. 9 non-English studies were retrieved but not screened due to limited access to translation services^10,12–19^. One study appeared to meet the inclusion criteria but was excluded because we could not confirm it was an independent sample of participants from a previously included study^20^. 37 studies were found to assume responsiveness but were not designed in a way to measure responsiveness (1 thoracic^21^, 23 lumbar^22–44^, 14 cervical^41,45–57^). The results of these studies (characteristics, risk of bias, and data summaries) have been included in the supplementary files as they may be useful for future work in this area. In total, 23 studies were included in this review, 4 of which assessed multiple measurement properties: 6 reliability (3 lumbar^58–60^, 3 cervical^61–63^), 21 discriminative validity (15 lumbar^22–24,59,60,64–73^, 6 cervical^45,61,62,74–76^), and 1 measurement error (lumbar^59^) (Fig. 1).

### Study characteristics

Across all measurement properties, the studies were conducted in Australia, Brazil, Canada, China, Hong Kong, Iran, Italy, Malaysia, Netherlands, New Zealand, Norway, Spain, South Korea, United Kingdom and USA. The majority of studies were collected in the laboratory setting. Study characteristics for reliability and measurement error, and discriminative validity are presented in Supplementary Tables S1A–C online.

### Risk of bias

**Figure 2:**
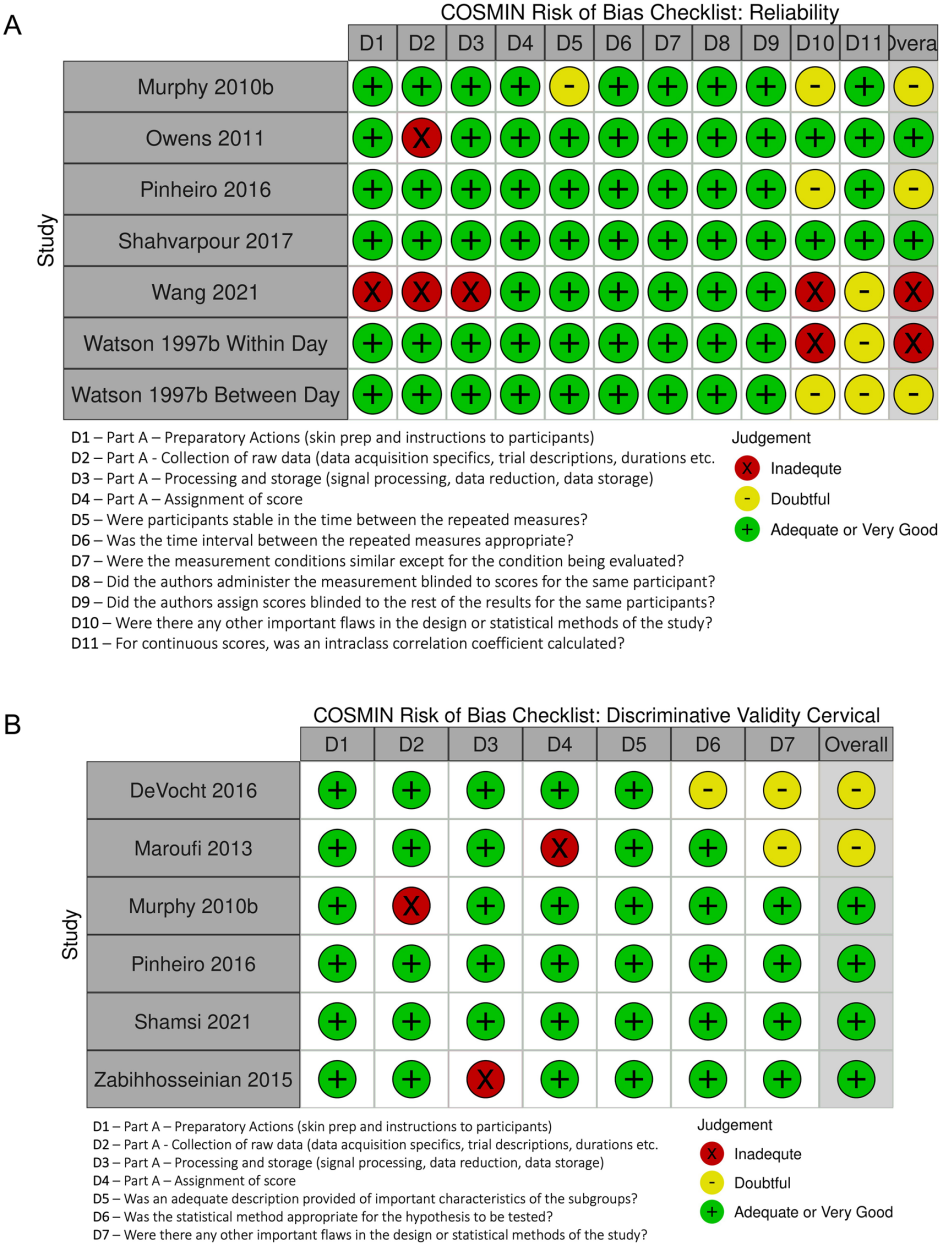

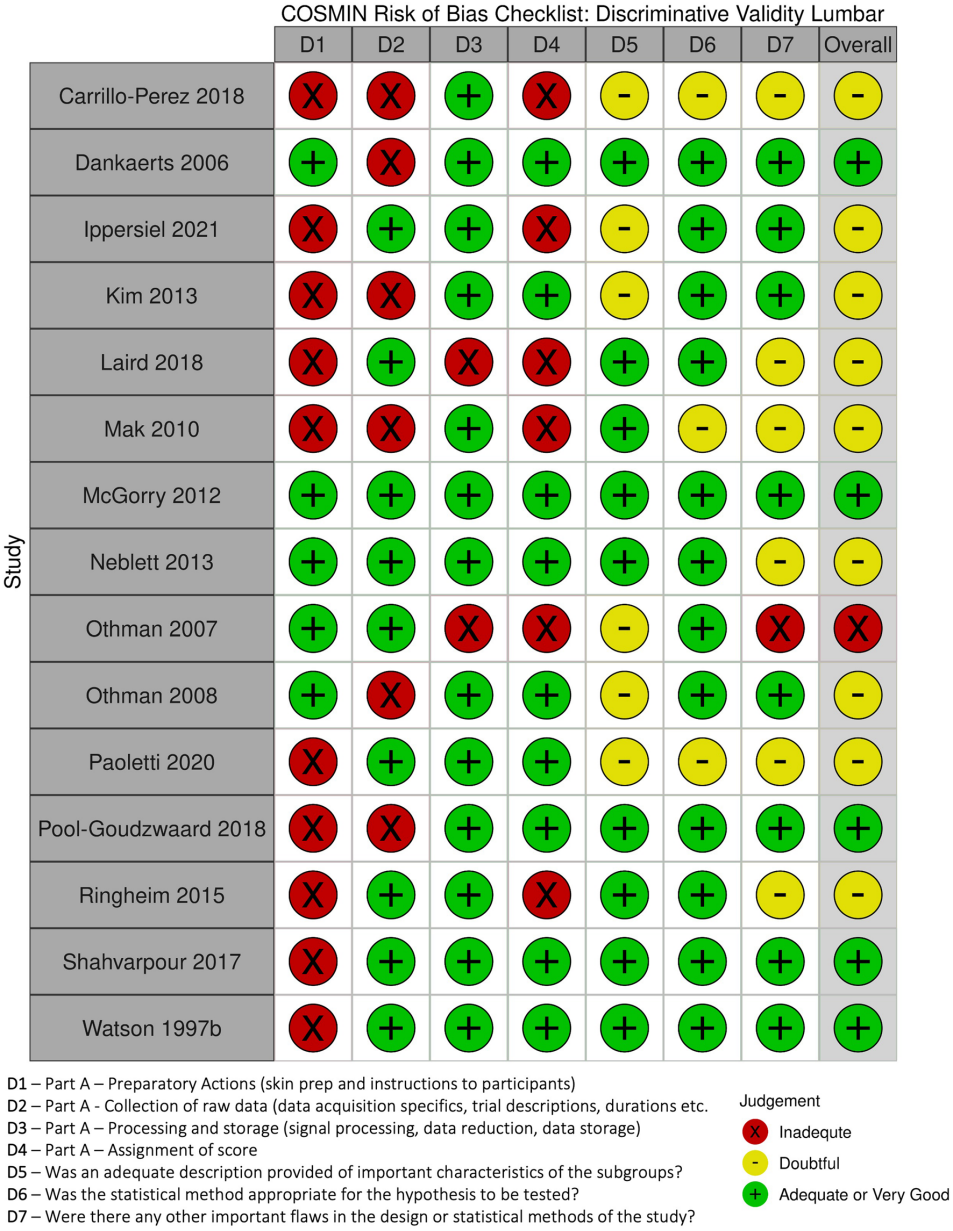
Individual domain (Part **A** and Part **B**) and overall risk of bias score for included studies that assessed (**A**) reliability, (**B**) cervical discriminative validity, and (**C**) lumbar discriminative validity.

For reliability, only two studies^58,59^ had overall risk of bias (RoB) ratings of adequate, three studies were rated doubtful (Watson 1997 for between-day reliability)^60–62^ and two studies were rated inadequate (Watson 1997 for within-day reliability)^60,63^ (Fig. 2A). The two domains that drove down the rating for the rest of the studies were D6 (Were there any other important flaws in the design or statistical methods of the study) and D7 (Statistical methods: not using the ICC for continuous scores). For discriminative validity, the majority^45,61,62,76^ of studies for the cervical FRR received adequate overall RoB scores with only two^74,75^ receiving overall ratings of doubtful (Fig. 2B). The majority^22,24,65–67,69,71–73^ of studies for the lumbar FRR received doubtful overall RoB scores with one receiving inadequate^70^, and five receiving adequate or very good overall scores^23,59,60,64,68^ (Fig. 2C).

### Individual studies

#### Reliability and meaurement error

**Figure 3:**
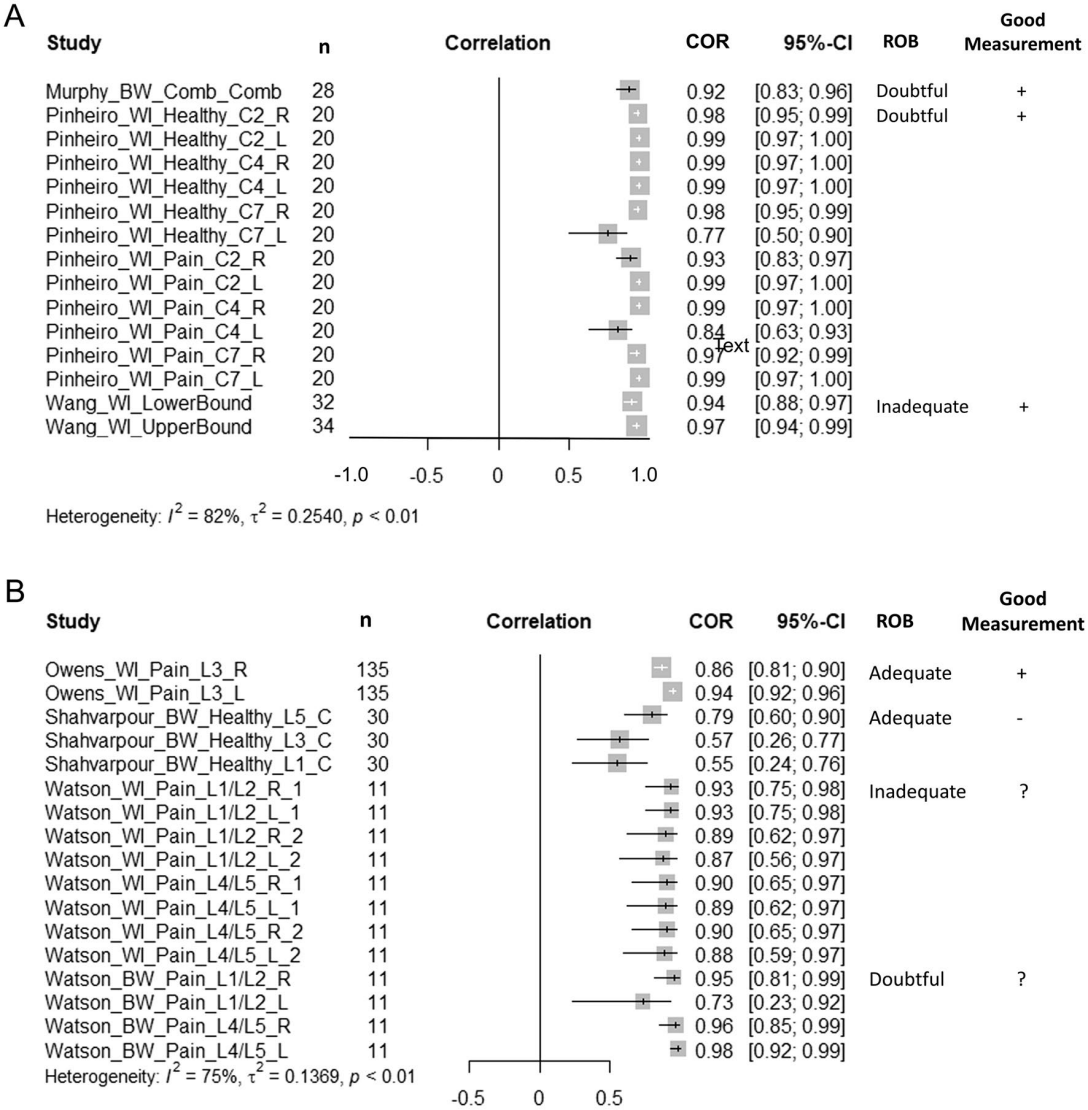
Forest plots of study findings for test–retest reliability of the flexion relaxation ratio (FRR) in the (**A**) cervical and (**B**) lumbar spine, including ROB and good measurement outcomes. CI, confidence interval; ROB, risk of bias.

The reliability results of individual studies are presented in Fig. 3A (cervical reliability) and Fig. 3B (lumbar reliability). The between-day reliability for the cervical FRR, assessed only by Murphy 2010 with a 4-week interval, was found to have excellent reliability (Intraclass correlation coefficients (ICCs); model not specified) ranging from 0.83 to 0.92^61^. Within-day reliability for the cervical FRR, assessed using ICC_1,2_^62^, ICC (model not specified^63^), and ICC_3,1_^58^ were found to be excellent overall (ICCs ranging from 0.84 to 0.99, with one 0.77). The between-day reliability of the lumbar FRR was assessed at intervals of 4 weeks^60^ and 8 weeks^59^. Between-day reliability in these studies was assessed with different statistics: generalizability theory framework dependability coefficient^59^ and Pearson’s correlation^60^ were variable, ranging from poor (0.55–0.57^59^) to excellent (0.87–0.98^60^). Within-day reliability of the lumbar FRR was higher, ranging from 0.86 to 0.94^58,60^.

Only one paper, Shahvarpour et al.^59^, calculated standard error of measurement (SEM). This was reported for the lumbar FRR for the multifidus, iliocostalis, and longissimus muscle groups. SEM was not substantially improved by repeated measurements and ranged from 1.8 to 3.9 across the three measurements per muscle.

#### Discriminative validity

**Figure 4:**
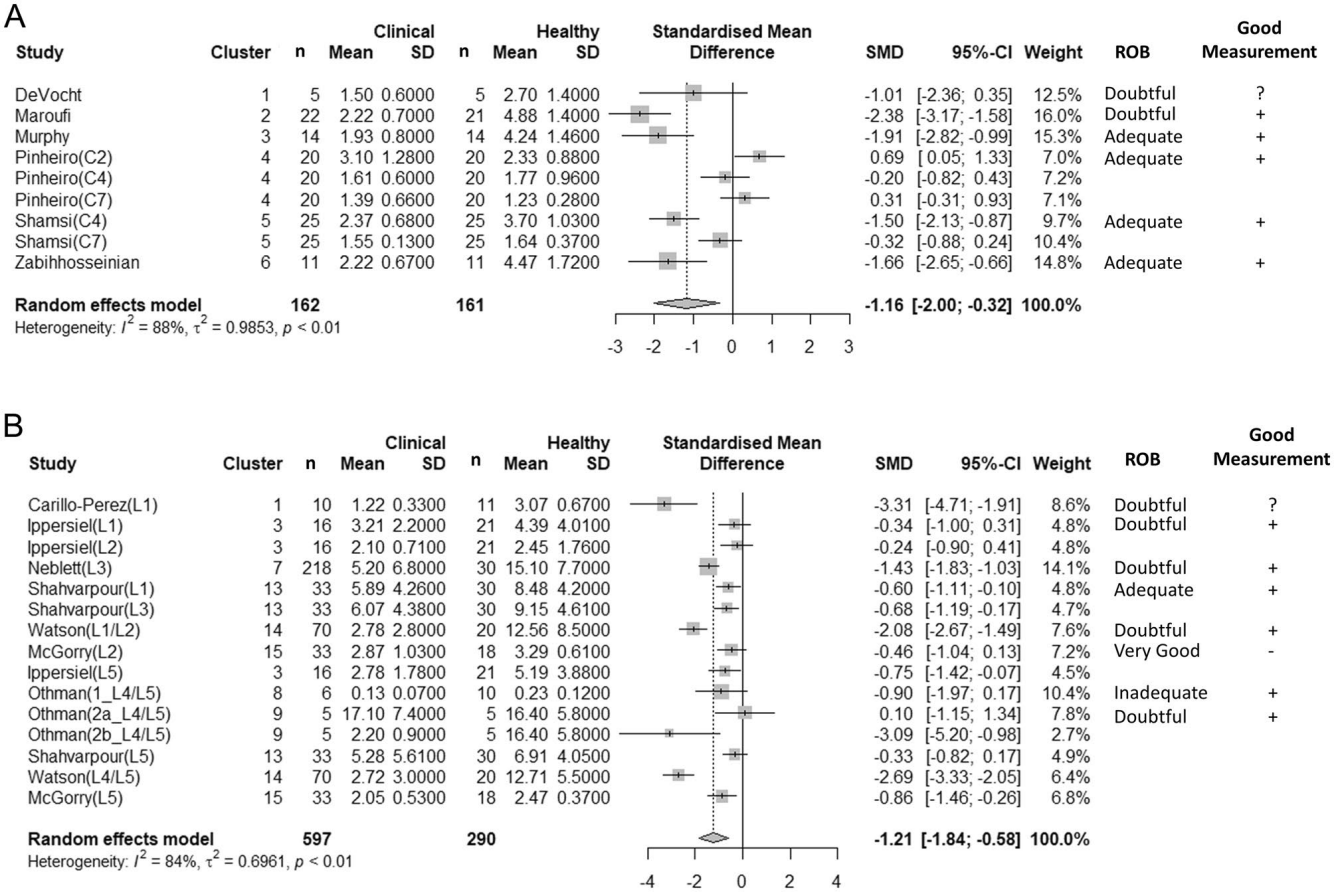
Forest plots of the standardized mean difference for discriminative validity of the flexion relaxation ratio (FRR) in the (**A**) cervical and (**B**) lumbar spine. Negative values indicate that the FRR was greater for the healthy group. Including ROB and good measurement outcomes. CI, confidence interval; ROB, risk of bias.

For the cervical FRR, most values were lower for clinical compared to healthy groups (Fig. 4A); however, we found that the 95% confidence interval (CI) for the standardized mean difference did not include zero in only 47.6% of the comparisons^45,61,62,75,76^.

Only studies that used a similar equation to calculate the cervical or lumbar FRR are summarized in Fig. 4A,B. There were several studies that compared the lumbar FRR between clinical and healthy groups, but used a different equation to calculate the FRR^22–24,31,64,67,72^. Data from these studies are summarized in Table 1.

**Table 1:** Results of discriminative validity of the lumbar flexion relaxation ratio (FRR) for studies using other equations to calculate the FRR.

Author	Equation	Location	Clinical			Healthy			Mean Diff	95% CI	ROB	Good Measure
			n	Mean	SD	n	Mean	SD				
Dankaerts, 2006	Sitting / Slumped Sitting	L5	33	0.95	1.01	34	1.38	1.01	− 0.43	[− 0.923, 0.063]	Very Good	+
		L1		1.09	0.98		1.38	1.00	− 0.29	[− 0.773, 0.193]		
Kim, 2013a	Full flexion / flexion* 100	L3/L4*	17	20.8	13.2	16	8.9	2.3	11.9	[5.068, 18.732]	Doubtful	+
		L3/L4^	14	24.5	14.4	16	8.9	2.3	15.6	[8.137, 23.063]		
Laird, 2018	Full flexion / flex + Ext	L3	140	0.25	0.32	124	0.012	0.32	0.238	[0.160, 0.316]	Doubtful	+
Mak, 2010	Upright Sitting / flexed sitting	L3	25	2.02	1.49	20	3.45	2.2	− 1.43	[− 2.542, − 0.318]	Doubtful	+
Paoletti, 2020	Full Flexion / Extension	L1, L5	12	0.55	0.26	13	0.25	0.2	0.3	[0.109, 0.491]	Doubtful	+
Pool-Goudzwaard, 2018	Extension / full flexion	L1, L4	16	1.41	0.39	24	1.5	0.33	− 0.09	[− 0.322, 0.142]	Adequate	+
Ringheim, 2015	NR	Bilateral ES	17	3.5	NR	20	10.3	NR	− 6.8	[− 16.98, 3.38]^ϕ^	Doubtful	+

SD, standard deviation; CI, confidence interval; ES, erector spinae, spine level not reported; NR, not reported.

^ϕ^95% CI estimated. Kim et al.^31^ subgroups; *LFRS; ^LERS.

Where authors present bilateral results, only R side reported. Paoletti and Pool-Goudzwaard presented combined results for bilateral measures at L1 and L5, and L1 and L4 respectively.

Similar to the cervical FRR, most values for the lumbar FRR were lower for the clinical compared to healthy groups (Fig. 4B, Table 1), and when considering the 95% CI for the standardized mean difference, we found this to be significant in 57.1% of the comparisons presented in the included papers^22,59,60,65–67,69,73^.

#### Inter-rater reliability of data from digitized graphs

A total of 170 data points were digitized from graphs in the included studies. Inter-rater reliability of digitized measurements was 0.99 with an average absolute value of the difference between raters of 6% (expressed relative to the average of measures between raters).

### Syntheses

The only measurement property that our review team felt could be adequately synthesized with a meta-analysis was the discriminative validity of the cervical and lumbar FRR for the studies that examined the same spine levels/muscles, with similar methods, and the same FRR equation. For the cervical FRR, the standardized mean difference of the pooled estimate favoured a lower FRR in people with neck pain (− 1.16 [− 2.00, − 0.32], *p* < 0.01, I^2^ = 88%, Fig. 4A). For the lumbar FRR, the standardized mean difference of the pooled estimate favoured a lower FRR in people with back pain (− 1.21 [− 1.84, − 0.58], *p* < 0.01, I^2^ = 84%, Fig. 4B).

### Certainty of evidence

Using the modified Grading of Recommendations Assessment, Development, and Evaluation (GRADE) criteria presented by the Consensus-Based Standards for the Selection of Health Measurement Instruments (COSMIN)^77^, this review concludes that there is moderate certainty of the evidence that there is high test–retest reliability for the cervical FRR and moderate to high test–retest reliability of the lumbar FRR; that there is high certainty of the evidence that the cervical and lumbar FRR can be used to discriminate between healthy and clinical groups (Table 2). This review was unable to draw a conclusion about the thoracic FRR or standard error of measurement (only one study each).

**Table 2:** Summary of findings for test–retest reliability, and discriminative validity for the cervical and lumbar flexion relaxation ratio (FRR).

Research question	What is the reliability, discriminative validity, responsiveness and measurement error of the FRR?			
Population	Adults (≥ 18 years old), either healthy (no recent history of LBP or clinical (experiencing episodes of LBP of any duration)			
Setting	Research or clinical settings			
Outcome attribute	Summary result	Overall rating (criteria for good measurement properties)*	Certainty of the evidence (GRADE)**	Comments
Reliability (test–retest) cervical	High test–retest reliability	+	Moderate ⊕ ⊕ ⊕Ο	Downgrade for Risk of Bias
Reliability (test–retest) lumbar	Moderate to high test–retest reliability	?	Moderate ⊕ ⊕ ⊕ Ο	Downgrade for inconsistency
Construct validity (discriminative validity) cervical	Can discriminate between healthy and clinical groups with a standardized mean difference of − 0.8211[− 1.8153, 0.1730]	+	High ⊕ ⊕ ⊕ ⊕	
Construct validity (discriminative validity) lumbar	Can discriminate between health and clinical groups with a standardized mean difference of − 1.21 [− 1.84, − 0.58]	+	High ⊕ ⊕ ⊕ ⊕	

*Good measurement property “+” = sufficient, “?” = indeterminant, “−” = insufficient.

**GRADE Rules:

Risk of Bias: No downgrade (multiple studies of at least adequate quality, or one study of very good quality), -1 Serious (multiple studies of doubtful quality or only one study of adequate quality), -2 Very serious (multiple studies of inadequate quality, or only one study of doubtful quality), -3 Extremely serious (only one study of inadequate quality).

Inconsistency: No downgrade (study results are consistent with each other or subgrouping explains inconsistent results/consistent results within subgroups), -1 Serious (result from at least one study is not consistent with the rest, with no explanation found, or only one study of adequate quality), -2 Very serious (results from more than one study are not consistent with the rest, with no explanation found). *In this case we may consider not pooling the data at all and not giving a quality level of the evidence.*

Imprecision: No downgrade (Total n > 100), -1 Total n = 50–100, -2 Total n =  < 50.

Indirectness: No downgrade (study population is well defined and matches our review inclusion criteria for population and clinical condition), -1 Serious (study population not well defined or representative of review population), -2 Very serious (evidence that the study population consists of individuals that would confound (misclassified/misrepresented) what is being studied).

## Discussion

This review found, with moderate certainty of the evidence that these studies show that the cervical FRR has high test–retest reliability and lumbar FRR has moderate to high test–retest reliability, and with high certainty of evidence demonstrating that the cervical and lumbar FRR can discriminate between healthy and clinical groups. There was not enough evidence to summarize findings for thoracic FRR discriminative validity or the standard error of measurement. We found numerous experimental studies that used the FRR as a dependent measure, but no studies were designed in a way that could confirm responsiveness.

Concerning reliability of the FRR outcome, only one study, Watson et al. 1997, was consistently identified between our study and previous reviews^60^. A study by Alschuler and colleagues was excluded from our review because their population included patients that had undergone spine surgery^78^. Marshall and Murphy 2006^79^ reported measures of reliability for the EMG signal magnitude in each phase of the forward bending movement task (i.e., flexing, flexed and extending phases), but was not considered for reliability in our review because they did not report reliability for the FRR. Overall, our review also found that test–retest reliability for the lumbar FRR was moderate to high, but with a moderate certainty of evidence because of inconsistency between studies. This, coupled with only a single study that reported measurement error^59^, underscores the need for further work on test–retest reliability if the lumbar FRR is to be considered as a biomarker for people with NSCLBP^3^.

The results of this review suggest that the FRR is a reliable outcome measure to use for the cervical and lumbar spine especially for within-session designs, however, one between-session study does show high reliability for between sessions (Murphy et al. 2010). Similarly, the review can confirm discriminative validity for the cervical and lumbar spine. Thus use of the FRR in the study of basic science questions pertaining to spine mechanics and function appear reasonable. However, without the ability to establish measurement error and responsiveness for the FRR we must conclude that there is currently limited clinical utility for this outcome measure.

There have been two recent reviews addressing the lumbar FRR and its potential use as a biomarker for people with NSCLBP^3,4^. The current review included a similar number of studies that reported on test–retest reliability for the FRR, but included a much larger number of studies (15) that compared between groups of participants with and without LBP. There are many methodological reasons that could have contributed to these discrepancies and it is difficult to identify precisely why these differences occurred. Both previous reviews concluded that the lumbar FRR had at least moderate test–retest reliability and was capable of discriminating between groups of people with and without NSCLBP, which is consistent with the findings of the current review. One strength of the current review over the previous reviews was our use of the modified GRADE approach defined by COSMIN that determined the certainties of the evidence for the level of test–retest reliability and discriminative validity^77^.

This review is the first, that we are aware of, to have systematically assessed the literature for measurement properties of the cervical FRR. The cervical FRR appeared to outperform the lumbar FRR for both test–retest reliability and discriminative validity with moderate and high certainty of evidence, respectively.

Neither of the previous reviews assessed responsiveness of the lumbar FRR; however, Moissenet and colleagues discussed the importance of this measurement property for clinical use of the lumbar FRR^3^. Responsiveness of a measurement has been referred to as “longitudinal validity” and defined as a measurement’s ability to detect change in a construct over time; however, the amount of measured change should be commensurate with changes in the construct (i.e., not disproportionately smaller or larger)^9^. Despite the search for this current review identifying many experimental studies that used the lumbar and cervical FRR as a dependent measure, none were considered adequate to assess responsiveness of the measurement. We identified two main issues that precluded the assessment of responsiveness. First was inconsistency across experimental studies at measuring changes in the construct that the FRR addressed. The second issue was related to the limited understanding of the test–retest reliability between-days and the associated between-day measurement error for the FRR^80^. Determining whether the FRR is responsive is one remaining gap in the literature.

Limitations of reporting were identified in Part A of the COSMIN risk of bias tool that rated the quality of reported preparatory actions, data collection, data processing/storage, and calculation of the final score. These items were not automatically used to determine the overall risk of bias for a study, but would indirectly impact one domain in Part B of the tool if an issue was identified that could impact “other important flaws in the study design”. Therefore, studies were downgraded for overall risk of bias if the authors either did not report the protocol for handling data from multiple trials (e.g., using the median value from a series of trials) or did not describe the process for combining data from muscles on the left and right sides of the body (e.g., averaging) when separate data from the left and right sides were not presented. This was captured in Part A by “calculation of the final score” and by “other” in Part B of the COSMIN RoB tool. A sensitivity analysis identified that 8 studies (35%) included in our review did not report the process for handling multiple trials or combining data between left and right sides, which affected the overall risk of bias rating in 4 of these studies (2 downgraded to adequate from very good, 1 downgraded to doubtful from very good and 1 downgraded to doubtful from adequate). Preparatory actions (e.g., removal of hair and cleaning the skin surface prior to electrode application) and details of the data collection (e.g., EMG amplifier characteristics and trial instructions) were also poorly described in many studies. These issues have recently been a focus of the Consensus for Experimental Design in Electromyography (CEDE) project that aims to improve consistency in acquisition, processing and reporting of EMG data in scientific studies^81,82^. A final limitation relates to the quantities that comprise the numerator and denominator of the FRR in many of the studies. Ratios are typically constructed with the quantity of interest in the numerator^83^. The characteristic of the FRP that distinguishes people with NSCLBP from control participants is the absence of a sudden decrease in the extensor EMG signal magnitude near the end range of forward flexion. Thus, the EMG signal magnitude in the fully flexed posture is the quantity of interest and should be presented in the numerator of the FRR; however, most studies calculate the FRR with this quantity in the denominator. Future work may consider calculating the FRR as the ratio of the average signal magnitude at full forward flexion divided by the peak signal magnitude during forward bending.

A recent study identified cut-off values, with high sensitivity and specificity for different methods of calculating the FRR and ERR to distinguish the presence and absence of the FRP in people with NSCLBP^84^. These authors reported many of the same issues with methodological consistency in formulating the FRR and ERR that were identified by our review. To better understand the utility of the FRR for use in clinical practice and research studies, future work should continue to investigate the test–retest reliability, measurement error, and responsiveness of the FRR. The large heterogeneity between studies may be improved with adherence to a consistent protocol for obtaining measures of the FRR. Examples of elements for the measurement protocol that should be standardized are: location and placement of electrodes, preparation of the electrode site, a standardized script of instructions to participants, pace of movements, post-collection processing of the EMG signals, and the method for extracting values that comprise the numerator and denominator of the FRR.

The current review included a robust team with content experts in surface EMG, a methodologist and a health sciences librarian. We followed the best-practices for a systematic review that included: registering the protocol, developing a sensitive search strategy that was peer-reviewed according to the Peer Review of Electronic Search Strategies (PRESS) guidelines, having independent raters for all stages with consensus, using COSMIN for evaluating RoB, assessing the certainty of evidence with GRADE, and reporting our findings in accordance with the Preferred Reporting Items for Systematic Reviews and Meta-Analysis (PRISMA) and the synthesis without meta-analysis (SWiM) guidelines. A potential limitation of this review was its scope, which led to the inclusion of many studies and division of the screening, data extraction, and RoB assessment across multiple pairs of reviewers. For example, two independent reviewers were each responsible for extracting information from half of the included studies with a third reviewer checking the extracted information for errors. Errors identified by the third reviewer were discussed with the person who completed the extraction to achieve consensus on the correct information. Multiple pairs of reviewers were responsible for independently completing the RoB assessment with a meeting to achieve consensus between the reviewers on the final determination. Again, a calibration exercise (pilot) was conducted for the risk of bias assessment to ensure consistency between pairs of reviewers. Our decision to use the COSMIN tool to assess RoB could be another limitation. This tool uses a very conservative “worst score principle” to determine the overall risk of bias for a study. Nonetheless, the COSMIN tool was chosen because of its specific focus on assessing the quality of evidence for an outcome/dependent measurement. Data extraction may have been limited by the use of a web-based tool for extracting data from digitized graphs that were presented in the included studies; however, inter-rater reliability for the digitized data obtained from graphs in the included studies of this review was near-perfect (ICC_2,1_ = 0.99) and is consistent with previously reported inter-rater reliability^85^. Our results are also limited by the quality of reporting in the source studies. For example, details of the study inclusion/exclusion critera for each group (clinical and healthy) were not always clearly outlined and outcome measures defining the characteristics of these groups (e.g., pain and disability) were not always measured or reported. Researchers should endeavour to strengthen these aspects of design and reporting in their studies. Finally, age may affect the FRR. Evidence from the literature suggests the FRR is lower in older (> 60 years of age) compared to younger (< 40 years of age)^86^. Concerning the impact of age of measurement properties of the FRR, however, there is some indication from data presented by Owens et al.^58^ that within-session reliability of FRR is not affected by age. However, the implications on measurement error and between-session reliability have yet to be explored for FRR. The data included in our synthesis represented a broad age range (young and older participants), which is a strength in that it is more generalizable. Specifically, we confirmed discriminative validity between groups despite the potential effects of age on FRR. Nonetheless, the potential confounding effects of age should be considered when interpreting these results and especially be considered in the design of future research studies in this area.

In conclusion, this review determined that the FRR and ERR have moderate-to-high test–retest reliability and can discriminate between people with either neck or low back pain and pain-free controls. The utility of these measurements in clinical practice and longitudinal research is limited by the lack of information regarding measurement error and responsiveness.

## Methods

### Registration and protocol

The protocol for this review was registered prior to the study start on Open Science Framework (https://doi.org/10.17605/OSF.IO/27EDF). We followed the working procedure for conducting a systematic review of validity, reliability and measurement error developed by COSMIN^9,77^. This report follows the most recent PRISMA guideline^87^.

### Eligibility criteria

#### Population

This review targeted studies that included adults (aged ≥ 18 years or older). Studies including both a clinical group and healthy group were included for the assessment of discriminative validity, while studies including a clinical group, healthy group, or heterogenous group were included for the assessment of reliability and responsiveness. A clinical group was defined in this review as participants currently experiencing an episode of non-specific spinal pain (i.e., neck, upper, or low back pain) of any duration (i.e., acute or persistent/chronic) and who were included in the study based on self-identification or an outcome measure of pain (e.g., visual analogue scale, numeric pain rating scale) or function/disability (e.g., Oswestry Disability Index). A healthy group was defined as participants with no current pain or recent history of non-specific spinal pain. We excluded data from clinical groups consisting of participants with a specific cause of spinal pain (e.g., infection, malignancy, fracture, history of spine surgery) or if the study authors did not screen for these potential causes of pain.

#### Outcome

We included studies that calculated the FRR from surface electromyography (sEMG) of spine muscles (i.e., neck, upper, or lower back) as a dependent measure. We deviated from our protocol to accept those with or without a concurrent measure of spine angle (e.g., motion capture, inertial motion sensor, or accelerometer/inclinometer) so long as the instructions for the FRR trial defined the motion of the trial (flexion and extension phases). Studies that employed all methods of calculation for the FRR were accepted (e.g., maximum root mean squared sEMG flexing divided by maximum root mean squared sEMG at full flexion, maximum root mean squared sEMG extending divided by maximum root mean squared sEMG at full flexion).

Included studies must have assessed the reliability or responsiveness of the FRR and/or discriminative validity of the FRR through hypothesis testing as or present enough data (mean and standard deviation per group) that could be used to assess validity or responsiveness against our review hypotheses. We used the data available that matched our inclusion criteria (i.e., if only the healthy/asymptomatic population matched our review inclusion criteria, and we could use that for reliability or responsiveness, it was included in those parts of the review respectively). Both populations were considered for studies of reliability or measurement error. Discriminative validity compared between populations that are known to be different^4^. Studies of responsiveness included either an experimental or clinical intervention applied to one or both populations.

#### Validity

Validity refers to the extent to which a measure assesses the construct it is supposed to measure^5^. Several aspects of validity need to be addressed when assessing the suitability of a measurement outcome. Construct validity is the aspect of validity that refers to the degree to which the scores of a measurement are consistent with hypotheses that align with the construct of interest^5^. For this review, the form of construct validity that we explored was discriminative validity, which refers to the ability of a measurement score to distinguish between predictably different individuals or groups. We hypothesized that clinical groups (i.e., individuals with non-specific spinal pain) will have significantly different FRR values (95% CIs for mean group differences do not overlap zero) compared to healthy/asymptomatic groups.

#### Reliability

Reliability refers to the extent to which scores are the same for repeated measurements and can be observed by different persons on the same occasion (inter-rater), over time (test–retest), or by the same person on different occasions (intra-rater) given that the value of the construct has remained stable^5^. The construct must be stable for evaluations of test–retest and intra-rater reliability. Measurement error, a component of reliability, refers to the systematic and random error of an observed score that is not attributed to true changes in the construct being measured^5^. Any measure of reliability was accepted for this review.

#### Responsiveness

Responsiveness refers to the ability of a measurement instrument to detect change over time, when there has been a change, such as in response to treatment or during progression of disease in the construct being measured^5^. To confirm responsiveness of the FRR outcome measure, we hypothesized that significant differences in FRR (95% CI for mean difference pre/post exposure do not cross zero) would be found before and after exposures/interventions.

#### Types of studies

We deviated from our protocol to only include articles published in peer-reviewed journals or full papers published in peer-reviewed conference proceedings. We included randomized controlled trials, cohort studies, case–control studies, cross-sectional studies, quasi-experimental studies, and laboratory experiments. No language limits were set. Attempts were made to translate studies to English for inclusion; however, in the event this was not possible, the identified studies were listed for future reviews to use. We excluded the following types of studies: feasibility studies, pilot studies, systematic and non-systematic reviews, protocols, theses/dissertations, commentaries, reports, and any other non-peer-reviewed studies.

#### Context

Studies conducted in either a clinical or laboratory setting were included.

### Information sources and search strategy

Six databases (MEDLINE via Ovid, Embase via Embase.com, CINAHL via EBSCO, SPORTDiscus via EBSCO, Web of Science Core Collection, and Scopus) were searched for published studies from inception to June 1, 2022. Search terms consisted of subject headings specific to each database (e.g., MeSH in MEDLINE) and free text words relevant to the search concepts, such as "flexion relaxation" and "spine". The search string was developed by content experts (DDC, SH, SM, MF) together with a health services librarian (KR) and the search strategy was peer-reviewed by a second librarian according to the PRESS guidelines^88^. The complete search strategies for all databases are included in Supplementary Information S2 online.

### Selection process

Results from each database were combined and imported into Covidence (Veritas Health Innovation, Melbourne, Australia) where duplicates were removed prior to screening. Results for each stage of the review were tracked in Microsoft Excel (Microsoft Corporation, Redmond, USA). For titles and abstracts the screening list was divided and two pairs of reviewers (DDC and SCM, DT and AS) independently screened each half for inclusion into the full text stage. Similarly, at the full text stage the list was divided into two and two pairs of reviewers (DDC and SCM, SH and AS) independently reviewed full texts for inclusion in the review. Reviewer pairs met at each stage for consensus and to resolve any discrepancies through discussion. A third reviewer (DDC or SH) was consulted frequently during the process to maintain consistency between review groups. At the full text stage consensus was achieved between the review groups through a separate meeting with DDC and SH where all of the full text articles and decisions were checked and confirmed. Backward citation tracking was conducted on all included studies. The reference lists of systematic reviews, pilot studies, feasibility studies, and protocols were screened for articles missed by our search (backwards search) and a forward search of all included articles was performed (articles citing the included articles were found and screened). Potential articles found by the backward and forward search were screened by AS and DDC and reviewed and confirmed by SH.

### Data collection process and data items

Data were extracted from the included articles by one reviewer and independently checked by a second reviewer. Discrepancies between reviewers were resolved through a consensus meeting. A third reviewer was available to resolve any discrepancies that could not be resolved. Available supplementary files were consulted during data extraction for any relevant data that was not directly presented in the original study. Study authors were contacted for clarification where necessary. Data extracted on the study populations included author, year, country, setting (i.e., clinical or laboratory), sample size, patient characteristics (e.g., age, location of pain, duration of pain, outcome measure used for inclusion into the study), and healthy population characteristics (e.g., age, definition). Information pertaining to the characteristics of the study investigators (e.g., professional background, level of training, and/or years of experience) was extracted where possible. Relevant methodological information included data collection and processing methods (e.g., equipment, preparatory action/instructions to participants, preparation of patients, unprocessed data collection, data processing and storage, and session information), description of the FRR calculation, measurement properties assessed, components repeated (for reliability), source(s) of variation varied (i.e., days, raters), and classification thresholds (if used). Data on the description of FRR calculation, FRR result (mean and variance), statistical analysis and results for each measurement property assessed in each relevant study were extracted and the criteria for good measurement properties were applied^9^. Data only displayed in graphs were extracted by one reviewer and checked by a second using Webplot Digitizer (Version 4.3, https://automeris.io/WebPlotDigitizer). Inter-rater reliability of digitized data was assessed using an ICC_2,1_ calculated with the *psych* package^89^ in R^90^. Standardized mean difference and 95% confidence intervals were calculated where possible (and not reported by the study authors). Final data tables were checked by a fourth person for errors.

### Risk of bias assessment and quality of reporting

Two pairs of reviewers independently assessed the quality of each included study using the COSMIN Risk of Bias (RoB) tools/checklists to assess reliability and measurement error, construct validity (Box 9b, discriminative validity) and responsiveness (Boxes 10b, 10c, 10d)^9^. A pilot of the assessment was conducted first, where reviewers independently assessed a sub-section of the included studies and then met to compare ratings for all domains within the RoB tool to confirm that the assessment procedure was being applied consistently by everyone. The COSMIN RoB tool and checklist are modular, meaning that the boxes in the tool and checklist were completed based on the measurement properties evaluated in each study. If a study reported multiple outcomes of one measurement property (e.g., inter-rater and intra-rater reliability), the corresponding box in the COSMIN RoB tool/checklist was completed more than once. Each standard within the COSMIN RoB box was rated as ‘very good’, ‘adequate’, ‘doubtful’, or ‘inadequate’ according to the criteria outlined by the tool. We followed the “worst score counts” principle, where the overall rating of the quality of each study was determined by taking the lowest rating of any of the standards in the COSMIN boxes used^9,77^. Reviewers met for consensus and a third reviewer helped to resolve discrepancies that could not be resolved through discussion. Quality of reporting was assessed through Part A of the COSMIN RoB tool, which was the same for all measurement properties and focused on reporting of the parameters specific to the data collection and analysis. Specifically, reporting of all details related to equipment (what was used to collect the data), preparatory actions (skin preparation and instructions to the participants), collection of the raw data (data acquisition specifics, trial descriptions, durations etc.), processing and storage (signal processing, data reduction, data storage) and the assignment of score (how FRR was calculated). If significant concerns were raised about how the data was collected, processed, or analyzed in Part A this was taken into consideration when scoring the “Other” domain of Part B (“were there any other important flaws in the design or statistical methods of the study”). We present the Part A results together with our results for RoB (Part B) in traffic-light plots (results of individual domains by study) prepared using ROBVIS^91^.

### Effect measures

Standardized mean difference and 95% confidence intervals were calculated where possible (and not reported by the study authors) for discriminative validity and responsiveness. A standard effect size was also calculated (change in the mean score divided by the standard deviation of the baseline) if responsiveness was not explicitly reported in a study but enough information was provided. There was no effect measure used for the synthesis of reliability and/or measurement error.

### Synthesis methods

Results of individual studies for each measurement property (e.g., the range of values, percentage of confirmed hypotheses) were summarized according to the COSMIN methodologies for systematic reviews. Specifically, the methodology for patient-reported outcomes^77^ was followed for discriminative validity, and the methodology for clinician-reported outcome measurement instruments, performance-based outcome measurement instruments, and laboratory values^9^ was followed for reliability, responsiveness, and measurement error. We checked study results against our review hypotheses for the assessment of construct validity and responsiveness.

Explanations for inconsistent results between studies for a measurement property (i.e., test–retest reliability) were explored and subgroups of homogeneous studies were summarized (e.g., different study populations, quality of the studies). If no explanation for inconsistency was found, we concluded that the results were inconsistent. Once again, the overall results were compared to the criteria for good measurement properties to determine whether FRR has sufficient (+), insufficient (−), or indeterminate (?) construct validity, reliability, measurement error, and/or responsiveness^9^. Results were reported by two reviewers for each measurement property. The reviewers met for consensus through discussion and a third reviewer helped to resolve persistent discrepancies.

Where possible, results from studies on reliability and discriminative validity were statistically pooled in a three-level random-effects meta-analysis using the package *meta* in R to account for multiple results from the same study^92,93^. Statistical heterogeneity was assessed by the I^2^ statistic per outcome. Only studies that reported confidence intervals (or from which we could calculate confidence intervals) and that used the same population, context, study design, FRR calculation, and statistical model/formula were quantitatively pooled and visualized with forest plots. The results of the remaining studies were presented in tables and a synthesis without meta-analysis was conducted in adherence with the SWiM Reporting Guidelines^94^.

### Certainty assessment: grading the quality of cumulative evidence

Two reviewers independently assessed the overall quality of evidence on validity, reliability, measurement error, and responsiveness of the FRR using the modified GRADE approach outlined by COSMIN methodology for systematic reviews of patient-reported outcome measures^9,77^. The quality of the evidence was graded as high, moderate, low, or very low evidence for our confidence in the measurement property estimates. Four factors were considered when evaluating the quality of the evidence: risk of bias (methodological quality of the studies); inconsistency (unexplained inconsistency of results across studies); imprecision (total sample size of the available studies); and indirectness (evidence from different populations than the population of interest). Each study started with the assumption that the overall result of the study was of high quality and could be downgraded by one to three levels based on each of these four factors. The rules for downgrading were presented in our protocol a priori. The two reviewers met for consensus and a third reviewer helped resolve any persistent discrepancies. The final grading of the quality of the evidence was recorded in a Summary of Findings Table together with the rules (table footnotes) and justifications for any decisions to downgrade.

### Supplementary Information

Supplementary Table 1A.

Supplementary Table 1B.

Supplementary Table 1C.

Supplementary Information 2.

Supplementary File 3a.

Supplementary Table 3b.

Supplementary Table 3c.

Supplementary Figure.

Supplementary Figure.

Supplementary Table 3f.

Supplementary Table 3g.

## Data Availability

All data generated or analysed during this study are included in this published article and its supplementary information files.
