# Supplementary material for: A systematic review and meta analysis of measurement properties for the flexion relaxation ratio in people with and without non specific spine pain
Source: Sci Rep. 2024 Feb 8;14:3260. doi: 10.1038/s41598-024-52900-z (PMC10853169; doi:10.1038/s41598-024-52900-z)
Supplement: Supplementary file 1 — Supplementary Table 1A. [file 41598_2024_52900_MOESM1_ESM.docx]

Supplementary Table 1A – Characteristics of included studies for flexion relaxation ratio (FRR) reliability and measurement error (cervical and lumbar combined). Mean (Standard Deviation) given unless otherwise stated. If Baseline and Analysis n are the same than only one value is included. The asterisk denotes that we have only included the pain group that was used for reliability testing. Measurement error was only reported by Shahvarpour et al., 2017. Abbreviations provided in footnote.

| **Author (Year); Country; Setting; Design; FRR Location; Reliability Type** | **Group** | **n** | **(I)nclusion, (E)xclusion Criteria** | **Age**  **(yr)** | **%F** | **Mass**  **(kg or lbs)** | **Height**  **(cm or m)** | **BMI**  **(kg/m^2^)** | **Duration**  **(mos or yr)** | **Outcome** |
| --- | --- | --- | --- | --- | --- | --- | --- | --- | --- | --- |
| Murphy  (2010b);  New Zealand;  Laboratory;  Cohort;  Cervical;  Test-retest between sessions | Pain | 14 | I: Chronic neck pain, defined as pain which persists for more than 3 months, aged 18-55 years, both genders. Had not received cervical adjustments or cervical exercises within the previous 3 months  E: NR | 44.8  (8.5) | 78.6 | 73.5  (14.4) | 1.67  (0.09) | 26.27  (5.3) | 8  (6) | NDI:  B – 18  (10)  VAS:  B – 37  (18) |
|  | Healthy | 14 | I: Participants aged 18-55 years, both genders, with very little to no neck pain. Had not experienced neck pain in the previous 3 months  E: NR | 27.3  (8.9) | 64.3 | 68.8  (11.3) | 1.72  (0.09) | 23.1  (2.6) | N/A | NDI:  B – 0.1  VAS:  B – 0 |
| Pinheiro  (2016);  Brazil;  Laboratory;  Cross-sectional;  Cervical;  Test-retest within session | Pain | 20 | I: Computer workers with chronic neck pain (at least 3 months), at least mild neck-related disability according to the Neck Disability Index (NDI), pain intensity of at least 3 on a numeric pain intensity scale (0-10) on most days, and working at the same job/position for at least 12 months  E: Have other acute or chronic pain disorders (MSK or not), systemic degenerative disease involving multiple joints, cervical whiplash syndrome and other traumatic conditions, congenital deformities of the upper limbs and spine, history of spine surgery, vision or hearing impairment, and cognitive deficits | 31.8  95% CI [30.2, 33.4] | 50 | 72.4  95% CI [64.5, 80.2] | 1.7  95% CI [1.6, 1.7] | 25.3  95% CI [23.5, 27.1] | 5.9  95% CI [4.3, 7.4] | Daily computer use (hrs):  B – 7.5  95% CI [7.1, 7.9]  NPRS (/10):  B – 4.5  95% CI [3.8, 5.2]  NDI:  B – 7.3  95% CI [6, 8.6] |
|  | Healthy  (Control) | 20 | I: No neck pain in past year and no score on the NDI. (20 who work with computers and 20 for a control group that reported using a computer for < 4h/day  E: Have other acute or chronic pain disorders (MSK or not), systemic degenerative disease involving multiple joints, cervical whiplash syndrome and other traumatic conditions, congenital deformities of the upper limbs and spine, history of spine surgery, vision or hearing impairment, and cognitive deficits | 28.3  95% CI [26.1, 30.5] | 50 | 63.4  95% CI [58.9, 67.8] | 1.7  95% CI [1.6, 1.7] | 22.3  95% CI [20, 23.7] |  | Daily computer use (hrs):  B – 1.5  95% CI [1.2, 1.8] |
|  | Healthy  (Computer use) | 20 | I: Same as Control, but work with computer for > 4h/day  E: Same as Control group | 29.5  95% CI [27.4, 31.6] | 50 | 73.57  95% CI [67.1, 80] | 1.7  95% CI [1.7, 1.8] | 24.8  95% CI [22.3, 26.3] |  | Daily computer use (hrs):  B – 7.5  95% CI [6.3, 8] |
| Wang  (2021);  China;  Laboratory;  Cross-sectional;  Cervical;  Test-retest within session | Pain (Cervical degeneration and malalignment) | 34 | I: Diagnosed with cervical spine degeneration with cervical malalignment, over 18 years old, BMI ≤ 30; Cobb angle (C2-C7) ≥ +4°  E: Neck pain for ≥ 1 year before study, VAS > 30 mm during the evaluation period, spine tumor, compression fracture, Parkinson disease, amyotrophic lateral sclerosis or other nerve system diseases, deficiency of normal lordosis or kyphosis, straight neck (Cobb C2-7 from -4° to + 4° | 44.7  (17.6) | 73 | NR | NR | 25.6  (3.1) | 7.7 mos  (2.6) | VAS:  B – 2.41  (1.15) |
|  | Pain (Normal cervical curvature) | 32 | I: Participants with normal cervical curvature matched to sex, age and BMI, recruited from same department as cases  E: NR | 42.5  (13.2) | 69 | NR | NR | 25.7  (3.4) | 6.5  (2.8) | VAS:  B – 1.59  (1.04) |
| Owens  (2011);  USA;  Laboratory;  Cross-sectional;  Lumbar;  Test-retest within session | Pain | 135 | I: Back-related leg pain > 3 (0-10 scale), Sub-acute or chronic back-related leg pain defined as current episode > 4wk duration, Back-related leg pain classified as 2,3,4, or 6 using the Quebec Task Force Classification system, this includes radiating pain into the proximal or distal part of the lower extremity, with or without neurological signs, with possible compression of a nerve root, 21 years of age and older, stable prescription medication plan (no changes in prescription medications that affect musculoskeletal pain in the previous month)  E: Ongoing treatment for leg or low back pain by other health care providers, progressive neurological deficits or cauda equina syndrome, QTF 1 (pain without radiation), 5 (spinal fracture), and 11 (other diagnoses including visceral diseases, compression fractures, metastases). QTF 7 (spinal stenosis syndrome characterized by pain and/or paresthesias in one or both legs aggravated by walking), uncontrolled hypertension or metabolic disease, blood clotting disorders, severe osteoporosis, inflammatory or destructive tissue changes of the spine, QTF 8 and 9 (surgical lumbar spine fusion) or patients with multiple incidents of lumbar surgery, QTF 10 chronic pain syndrome, pregnant or nursing women, current or pending litigation, inability to read or verbally communicate in English, evidence of narcotic or other drug abuse, unwillingness to postpone all other types of manual therapy treatment for LBP or BRLP except those provided in the study for the duration of the study period | 57.20  (12.04) | NR | 173.68  (33.79) | 66.25  (3.44) | 27.79  (4.86) | NR | NPRS_back_ (/10):  B – 5.36  (2.22)  NPRS_leg_ (/10):  B – 5.48  (1.67)  RMDQ (/23):  B – 10.32  (5.07)  FABQ-W (/30):  B – 9.79  (7.94)  FABQ-PA (/30):  B – 10.68  (5.26) |
| Shahvarpour  (2017);  Canada;  Laboratory;  Cohort study;  Lumbar;  Test-retest within session | Pain | 33 | I: 18-65 years old adults who speak English or French and have lumbar or lumbosacral pain (with or without radicular pain) for at least 4 weeks (non-acute phase); and a score higher than 12% on the Oswestry Disability Index (ODI).  E: BMI > 30 kg/m^2^, prior surgery of the pelvis or spinal column, scoliosis, systemic or degenerative disease, a positive response to the Physical Activity Readiness Questionnaire, history of neurological diseases or deficits not related to back pain (e.g. Stroke, peripheral neuropathies and balance deficits), use of anticonvulsive, antidepressive and anxiolytic medication (use of antispasmodic, anti-inflammatory and analgesic drugs for back pain were accepted), pregnancy, claustrophobia, abnormal arterial blood pressure (hypertension). | M:  44.5  (13.9)  F:  47.8  (12.2) | 47.8 | M:  76.5  (13.2)  F:  71.5  (10.1) | M: 172.2  (6.4)  F:  163.1  (5.9) | M:  25.8  (3.9)  F:  26.8  (3.1) | NR | M:  ODI (%):  B – 26.4  (8.2)  NPRS (/10):  B – 5.0  (1.7)  F:  ODI (%):  B – 30.8  (10.2)  NPRS (/10):  B – 4.8  (1.0) |
|  | Healthy | 30 | I: 18-65 years old adults who speak English or French  E: Same as pain population | M:  39.3  (14.3)  F:  39.8  (14.1) | 50 | M:  77.1  (10.1)  F:  62.9  (10.6) | M:  178.1  (8.4)  F:  164.2  (5.9) | M:  24.4  (3.2)  F:  23.3  (3.6) | N/A | N/A |
| Watson  (1997);  United Kingdom;  Laboratory;  Cross-sectional;  Lumbar;  Test-retest within and between sessions | Pain* | 11 | I: Chronic low back pain as the primary presenting condition, duration of at least 6 months, not undergoing any current treatment other than routine analgesia, willingness to participate in a pain management program, aged between 18 and 65 years.  E: Major structural abnormality (e.g., kyphoscoliosis), evidence of inflammatory, systemic or neoplastic disease, major psychiatric illness, pregnancy, and any other medical condition likely to interfere with an active rehabilitation program. | 39.4  (6.9) | 55 | NR | NR | NR | 4.4  (4.1) | NR |

A = Analysis, B = Baseline, BMI = Body Mass Index, E = Exclusion Criteria, F = Females, FABQ = Fear Avoidance Beliefs Questionnaire, FABQ-PA = Fear Avoidance Beliefs Questionnaire for physical activity, FABQ-W = Fear Avoidance Beliefs Questionnaire for work, I = Inclusion Criteria, M = Males, N/A = Not applicable, NDI = Neck Disability Index, NPRS = Numeric Pain Rating Scale, NR = Not Reported, ODI = Oswestry Disability Index, RMDQ = Roland Morris Disability Questionnaire, VAS = Visual Analog Scale, 95%CI = 95 Percent Confidence Interval.
