# Supplementary material for: A systematic review and meta analysis of measurement properties for the flexion relaxation ratio in people with and without non specific spine pain
Source: Sci Rep. 2024 Feb 8;14:3260. doi: 10.1038/s41598-024-52900-z (PMC10853169; doi:10.1038/s41598-024-52900-z)
Supplement: Supplementary file 2 — Supplementary Table 1B. [file 41598_2024_52900_MOESM2_ESM.docx]

Supplementary Table 1B – Characteristics of included studies for discriminative validity of the cervical flexion relaxation ratio (FRR). Mean (Standard Deviation) given unless otherwise stated. If Baseline and Analysis n are the same than only one value is included.

| **Author (Year); Country; Setting; Design** | **Group** | **n** | **(I)nclusion, (E)xclusion Criteria** | **Age**  **(yr)** | **%F** | **Mass**  **(kg)** | **Height**  **(cm or m)** | **BMI**  **(kg/m^2^)** | **Duration**  **(mos or yr)** | **Outcome** |
| --- | --- | --- | --- | --- | --- | --- | --- | --- | --- | --- |
| DeVocht (2016);  USA; Laboratory & Pain Clinic; Cross-sectional | Pain | B: 6  A: 5 | I: Same as Healthy population with addition of current neck pain at least 2/10 on numerical rating scale  E: Same as Healthy population | 52.8  (11.1) | 67 | NR | NR | NR | NR | NPRS:  B – 5.4  (2.1) |
|  | Healthy | B: 6  A: 5 | I: Between 21-75 years old, good general physical condition by self-report, ambulatory and self-sufficient, no current neck pain  E: Pacemaker, artificial implants in cervical spine, fractures in upper trunk or cervical spine, other major health issues such as cancer, neurological diseases, progressive neurological disorders, ticks, spasms, taking medication for neck pain other than non-prescription analgesics | 34.2  (14.5) | 0 | NR | NR | NR | N/A | N/A |
| Maroufi (2013);  Iran; Laboratory; Cross-sectional | Pain | 22 | I: Diagnosis of non-specific chronic neck pain, persistent pain for more than 3 mos without significant pathology (e.g., fracture, mechanical instability, radiculopathy, myelopathy)  E: Pain > 30mm on VAS during assessment, spinal or shoulder trauma, surgery or systemic disease, strengthening exercise programs for the neck muscles | 23.45  (2.6) | 100 | 55.36  (4.3) | 162.41  (2.4) | 21  (1.7) | 2.09  (0.6) | VAS_pain_ (mm):  B – 20.9  (7.5) |
|  | Healthy | 21 | I: NR  E: Neck pain 1 year prior to study, spinal or shoulder trauma, surgery, or systemic disease, strengthening exercise programs for the neck muscles | 23.48  (1.8) | 100 | 56.24  (6.2) | 162.86  (1) | 21.2  (2.2) | N/A | N/A |
| Murphy  (2010b); New Zealand; Laboratory; Cohort | Pain | 14 | I: Chronic neck pain, defined as pain which persists for more than 3 months, aged 18-55 years, both genders. Had not received cervical adjustments or cervical exercises within the previous 3 months.  E: NR | 44.8  (8.5) | 78.6 | 73.5  (14.4) | 1.67  (0.09) | 26.27  (5.3) | 8  (6) | NDI (/50):  B – 18  (10)  VAS (mm):  B – 37  (18) |
|  | Healthy | 14 | I: Participants aged 18-55 years, both genders, with very little to no neck pain. Had not experienced neck pain in the previous 3 months.  E: NR | 27.3  (8.9) | 64.3 | 68.8  (11.3) | 1.72  (0.09) | 23.1  (2.6) | N/A | NDI (/50):  B – 0.1  VAS (mm):  B – 0 |
| Pinheiro (2016);  Brazil; Laboratory; Cross-sectional | Pain | 20 | I: Computer workers with chronic neck pain (at least 3 months), at least mild neck-related disability according to the Neck Disability Index (NDI), pain intensity of at least 3 on a numeric pain intensity scale (0-10) on most days, and working at the same job/position for at least 12 months  E: Have other acute or chronic pain disorders (MSK or not), systemic degenerative disease involving multiple joints, cervical whiplash syndrome and other traumatic conditions, congenital deformities of the upper limbs and spine, history of spine surgery, vision or hearing impairment, and cognitive deficits | 31.8  95% CI [30.2, 33.4] | 50 | 72.4  95% CI [64.5, 80.2] | 1.7  95% CI [1.6, 1.7] | 25.3  95% CI [23.5, 27.1] | 5.9  95% CI [4.3, 7.4] | Daily computer use (hrs):  B – 7.5  95%CI [7.1, 7.9]  NPRS (/10):  B – 4.5  95%CI [3.8, 5.2]  NDI:  B – 7.3  95% CI [6, 8.6] |
|  | Healthy  (Control) | 20 | I: No neck pain in past year and no score on the NDI. (20 who work with computers and 20 for a control group that reported using a computer for < 4h/day  E: Have other acute or chronic pain disorders (MSK or not), systemic degenerative disease involving multiple joints, cervical whiplash syndrome and other traumatic conditions, congenital deformities of the upper limbs and spine, history of spine surgery, vision or hearing impairment, and cognitive deficits | 28.3  95% CI [26.1, 30.5] | 50 | 63.4  95% CI [58.9, 67.8] | 1.7  95% CI [1.6, 1.7] | 22.3  95% CI [20, 23.7] | N/A | Daily computer use (hrs):  B – 1.5  95%CI [1.2, 1.8] |
|  | Healthy  (Computer use) | 20 | I: Same as Control, but work with computer for > 4h/day  E: Same as Control group | 29.5  95% CI [27.4, 31.6] | 50 | 73.57  95% CI [67.1, 80] | 1.7  95% CI [1.7, 1.8] | 24.8  95% CI [22.3, 26.3] | N/A | Daily computer use (hrs):  B – 7.5  95%CI [6.3, 8] |
| Shamsi (2021);  Iran; Laboratory; Cross-sectional | Pain | 25 | I: Diagnosed with non-specific chronic neck pain by an orthopedist. Persisted pain lasting for at least 3 months without any specific pathology (no history of degenerative disease, discopathy, radiculopathy)  E: Current pain score > 50 mm on Visual Analog Scale (VAS), systemic disease, cervical or shoulder trauma, or participated in neck muscle strengthening exercises in the past 3 months | 32.64  (5.41) | 40 | 69.64  (4.79) | NR | 23.68  (1.75) | 12mos IQR [8, 14] | VAS (mm):  B – 30 (median)  IQR [25, 30] |
|  | Healthy | 25 | I: Not experienced neck, head, shoulder, or low back pain at least within the last year  E: NR | 30.64  (4.40) | 52 | 69.96  (6.87) | NR | 23.86  (1.6) | N/A | N/A |
| Zabihhosseinian (2015);  Canada; Laboratory; Cross-sectional | Pain | B: 12  A: 11 | I: Mild (NDI scores of 5-14) to moderate (NDI scores of 15-24) neck pain for at least 3 months  E: NR | 23.1  (3.81) | 58 | 73.1  (21.57) | 168.75  (15.38) | NR | 3  (2) | Repetition of neck pain (#/wk):  B – 4  (2)  NDI:  B – 9.75  (3.88) |
|  | Healthy | B: 13  A: 11 | I: Free from chronic or recurrent neck, shoulder or elbow pain for at least 3 months prior (NDI scores of 0-4)  E: NR | 25.76  (4.51) | 46 | 66.15  (13.41) | 168.69  (8.91) | NR | N/A | N/A |

A = Analysis, B = Baseline, E = Exclusion Criteria, F = Females, I = Inclusion Criteria, IQR = Interquartile Range, N/A = Not Applicable, NDI = Neck Disability Index, NPRS = Numeric Pain Rating Scale, NR = Not Reported, ODI = Oswestry Disability Index, VAS = Visual Analog Scale, 95%CI = 95 Percent Confidence Interval.
