# Supplementary material for: A systematic review and meta analysis of measurement properties for the flexion relaxation ratio in people with and without non specific spine pain
Source: Sci Rep. 2024 Feb 8;14:3260. doi: 10.1038/s41598-024-52900-z (PMC10853169; doi:10.1038/s41598-024-52900-z)
Supplement: Supplementary file 3 — Supplementary Table 1C. [file 41598_2024_52900_MOESM3_ESM.docx]

Supplementary Table 1C – Characteristics of included studies for discriminative validity of the lumbar flexion relaxation ratio (FRR). Mean (Standard Deviation) given unless otherwise stated. If Baseline and Analysis n are the same than only one value is included.

| **Author (Year); Country; Setting; Design** | **Group** | **n** | **(I)nclusion, (E)xclusion Criteria** | **Age**  **(yr)** | **%F** | **Mass**  **(kg)** | **Height**  **(cm or m)** | **BMI**  **(kg/m^2^)** | **Duration**  **(mos or yr)** | **Outcome** |
| --- | --- | --- | --- | --- | --- | --- | --- | --- | --- | --- |
| Carillo-Perez (2018);  Spain; Laboratory; Cross-sectional | Pain | 10 | I: NR  E: NR | 37.33  (9.05) | NR | 64.75  (7.17) | 1.67  (0.054) | NR | NR | NR |
|  | Healthy | 11 | I: NR  E: NR | 26.70  (6.59) | NR | 70.30  (7.16) | 1.736  (0.11) | NR | N/A | N/A |
| Dankaerts (2006);  Australia; Orthopaedic Clinic;Cross-sectional | Pain  (FP) | 20 | I: >3 months nonspecific LBP; Revised Oswestry score >15%; Pain localized to the lower lumbar spine (L4–L5 or L5–S1) region; Absence of “red flags” (specific causes of LBP such as cauda equina syndrome or inflammatory disease); Absence of dominant “yellow flags” (identification of beliefs, emotions, and behaviors that interact with the pain problem); Clear mechanical basis of disorder; Associated impairments in the control of the motion segment(s) in the provocative movement direction(s); Absence of impaired movement of the symptomatic segment in the painful direction of movement or loading (based on clinical joint motion palpation examination); Clinical diagnosis of an FP or AEP disorder, both clinicians (independently) agreed upon the diagnosis  Key clinical features of FP: Aggravation of symptoms with movements and postures involving  flexion of the lower lumbar spine; Loss of segmental lordosis at symptomatic level, difficulty assuming and/or maintaining neutral lordotic postures with a tendency to flex lower lumbar spine; Pain relief with spinal extension  E: Previous spine surgery; Pregnant at the time of the study or 6 months postpartum; Recently undergone a period of motor control  Rehabilitation; Not fulfilling inclusion criteria | 35.7  (11.2) | 20 | 80.1  (10.6) | 1.8  (0.1) | 24.6  (2.5) | 4.9  (5.3) | R-ODI (%):  B – 36.6  (11.0) |
|  | Pain  (AEP) | 13 | I: Same as FP aside from key clinical features.  Key clinical features of AEP: Aggravation of symptoms with movements and postures involving  extension of the lower lumbar spine; Excess of segmental lordosis at symptomatic level with posture and movements; Difficulty assuming and/or maintaining neutral lordotic postures with a tendency to position themselves into hyperextension; Pain relief with spinal flexion  E: Same as FP. | 39.9  (11.3) | 61.5 | 72.8  (15.7) | 1.70  (0.1) | 24.2  (2.8) | 7.4  (5.3) | R-ODI (%):  B – 41.2  (14.2) |
|  | Healthy | 34 | I: Students; Relatives of students; University personnel  E: History of LBP or leg pain in previous 2 years; Received previous postural education | 32.0  (12.2) | 47 | 68.4  (11.6) | 1.71  (0.09) | 23.3  (2.9) | N/A | N/A |
| Ippersiel (2021);  Canada; Laboratory; Cross-sectional | Pain | 16 | I: Subacute or chronic non-specific low back pain, 18-49 years old, experiencing a pain episode, pain primarily in low back, no signs of serious underlying condition (e.g., cancer), spinal stenosis, or radiculopathy, or another specific cause of spinal pain (e.g. fracture), current pain intensity ≥ 2/10, current score on Oswestry Disability Index ≥ 13  E: NR | 30  (9) | NR | NR | NR | NR | 109.9 mos  (113.5) | NPRS (/10):  B – 3.4  (1.1)  ODI (%):  B – 25.3  (7.4)  STarTBack (/9):  B – 4.4  (1.8) |
|  | Healthy | 21 | I: No history of low back pain that limited activities  E: NR | 27  (10) | NR | NR | NR | NR | N/A | N/A |
| Kim (2013);  South Korea; Laboratory; Cross-sectional | Pain  (LFRS) | 17 | I: Mechanical low back pain without radiating pain  E: History of spinal or leg surgery, diagnosis of ankylosing spondylitis, diagnosis of rheumatoid arthritis, diagnosis of degenerative disease, diagnosis of any other neurological disorder, visually fixed kyphosis or scoliosis | 23.5  (2.4) | NR | 67.2  (11.9) | 173.3  (9.8) | NR | NR | NR |
|  | Pain  (LERS) | 14 | I: Same as LFRS group  E: Same as LFRS group | 23.8  (3.9) | NR | 65.0  (11.2) | 169.4  (7.0) | NR | NR | NR |
|  | Healthy | 16 | I: No history of low back pain  E: NR | 23.8  (2.9) | NR | 61.3  (9.2) | 169.1  (8.7) | NR | N/A | N/A |
| Laird (2018);  Australia; Laboratory; Cross-sectional | Pain | 140 | I: 18-65 years old, low back pain > 3mos, pain score ≥3/10, current back pain with or without leg pain  E: Previous lumbar surgery, any invasive spinal procedures for low back pain within last 12 mos (including therapeutic injections), serious medical or musculoskeletal issues that could affect lumbopelvic region, implanted electric medical device, BMI > 30, pregnancy | 41.4  (12.6) | 57 | NR | NR | 25.6  (4.9) | NR | Pain Intensity (0-10):  B – 5.3  (1.5)  RMDQ (0-100):  B – 39  (21)  FLAG (0-48):  B – NR |
|  | Healthy | 126 (B)  124 (A) | I: No significant health issues that would affect movement, no history of low back pain that required visiting a health professional or taking time off either work or usual sport  E: Back pain at the time of testing, an episode of back pain that had necessitated attending a medical practitioner or allied health professional in the last 12 months, time off work due to back pain in the last 12 months or, any back pain during or between testing procedures. (*Authors reference Laird BMC Musculoskelet Disord 17 403 2016*) | 34.4  (13.5) | 59 | NR | NR | 23.6  (3.5) | N/A | N/A |
| Mak  (2010); Hong Kong; Laboratory; Cohort | Pain | 25 | I: Back pain > 3 months  E: Prior back surgery, clinically identifiable pathology (e.g., herniated disc), nerve root compression, spinal stenosis, spondylolisthesis, inflammatory arthritis, cancer, pregnant, medical condition that could be exacerbated by participation | 42.2  (10.56) | 12 | NR | NR | 24.0  (5.6) | NR | ODI (%):  B – 46.64  (10.09)  F – 41.58  (13.51)  VAS_rest_ (/100):  B – 31.16  (18.50)  F – 31.56  (18.56)  VAS_exertion_ (/100):  B – 68.88  (15.46)  F – 66.96  (18.53) |
|  | Healthy | 20 | I: No history of significant back pain  E: NR | 29.2  (4.75) | 15 | NR | NR | 25.3  (2.6) | N/A | N/A |
| McGorry  (2012); USA; Laboratory;  Cross-sectional | Pain | 33 | I: 18-65. Currently experiencing backpain (new episode or recurrent)  E. Major structural abnormalities, significant neurological deficits or evidence of severe nerve root compression, active systemic, inflammatory, musculoskeletal or neoplastic disease or history of previous back surgery. Active worker’s compensation claim or related litigation pending. | 40.5 (12.8) | 48 | 75 (15.3) | 168.9 (10.4) | NR | 7.3 yr (7.5) | NPRS (/10): 3.0 (SD 1.6)  BPFS (/60): 43.9 (8.0) |
|  | Healthy | 18 | I: 18-65 years old. In good health and no significant history of back pain.  E: NR | 35.2 (9.4) | 44 | 69.7 (11.7) | 168.4 (10.5) | NR | N/A | N/A |
| Neblett (2013);  USA; Laboratory; Cohort | Pain | 218 | I: Patients with chronic low back pain (CLBP), with compensable occupational lumbar injuries under the Texas Worker's Compensation system, who were enrolled in an interdisciplinary functional restoration rehabilitation program and had demonstrated a minimum 4 months of disability since their injuries  E: NR | 46.8  (9.5) | 35.8 | NR | NR | NR | 28.4 mos  (34.7) | VAS_intensity_ (/10):  B – 7.1  (1.6)  MVADS:  B – 106.3  (17.6)  TSK (/68):  B – 33.8  (7.2)  BDI:  B – 22.9  (12.2) |
|  | Healthy |  | I: No history of low back pain over the prior year, no prior low back disability, no previous low back surgery, and no evidence of a gross scoliosis curve that might alter myoelectric behaviors.  E: NR | 37.6  (9.3) | 43.3 | NR | NR | NR | N/A | N/A |
| Othman (2007);  Malaysia; Laboratory; Cross-sectional | Pain | 6 | I: Female patients aged between 20-55, with LBP, for at least 12 weeks, that had not suffered from back pain due to a non-musculoskeletal disorder  E: Pregnancy | 35.67  (12.56) | 100 | NR | NR | NR | NR | NR |
|  | Healthy | 10 | I: Healthy females aged between 20-55.  E: Pregnancy | 24  (1.49) | 100 | NR | NR | NR | N/A | N/A |
| Othman (2008);  Malaysia; Laboratory; Cross-sectional | Pain | 5  (FRP+) | I: Adult females between the ages of 20-50 years old experiencing LBP for at least 12 weeks.  E: Pregnancy, suffered LBP due to a non-musculoskeletal disorder. | 29  (8) | 100 | NR | NR | 22.6  (3.1) | NR | NR |
|  | Pain | 5  (FRP-) | I: Same as FRP+ group  E: Same as FRP+ group | 37  (11) | 100 | NR | NR | 25.9  (8) | NR | NR |
|  | Healthy | 5 | I: "Healthy" adult females between the ages of 20-50 years old  E: Pregnancy | 28  (8) | 100 | NR | NR | 20.0  (0.9) | N/A | N/A |
| Paoletti (2020);  Italy; Laboratory; Cross-sectional | Pain | 12 | I: Aged between 18-65 years old, available to participate in a pain management program, actively suffering from LBP (LBP type should be specified, and it should also be clarified whether it is present when the test is executed).  E: Severe structural deformities (e.g., kyphoscoliosis), systemic diseases (a disease that affects multiple apparatuses or organs, often related to rheumatic diseases, or rare diseases such as genetic disorders) or neoplastic diseases (tumours), significant psychiatric diseases, pregnancy, any other medical condition that could interfere with the correct execution of the protocol. | 51  (8.21) | 42 | NR | NR | NR | NR | NR |
|  | Healthy | 13 | I: Aged 18-65 years old, no history of musculoskeletal or abdominal pain, not under medical treatment, no episodes of LBP within the last 6 months, no consultation with a therapist or doctor regarding LBP problems.  E: Severe structural deformities (e.g., kyphoscoliosis), systemic diseases (a disease that affects multiple apparatuses or organs, often related to rheumatic diseases, or rare diseases such as genetic disorders) or neoplastic diseases (tumours), significant psychiatric diseases, pregnancy, any other medical condition that could interfere with the correct execution of the protocol . | 39  (13.18) | 54 | NR | NR | NR | N/A | N/A |
| Pool-Goudzwaard (2018);  Netherlands; Laboratory; Cross-sectional | Pain | 16 | I: Age between 20-60 years old, being able to read and understand Dutch.  E: Specific LBP due to malignant processes and systematic disease as well as the inability to bend forward. | 37  (11.9) | 50 | NR | NR | NR | NR | RMDQ (/24):  B – 5.2  (4)  NPRS (/10):  B – 4  (2.5) |
|  | Healthy  (Study 1) | 24 | I: Age between 20-60 years old, being able to read and understand Dutch.  E: NR | 34  (12.3) | 46 | NR | NR | NR | N/A | RMDQ (/24):  B – 0  NPRS (/10):  B – 0 |
|  | Healthy  (Study 2) | 6 | I: Same as Study 1 group  E: Same as Study 1 group | 27  (11.9) | 67 | NR | NR | NR | N/A | RMDQ (/24):  B – 0  NPRS (/10):  B – 0 |
| Ringheim (2015);  Norway; Laboratory; Cross-sectional | Pain | 17 | I: Diagnosed with chronic LBP for more than 3 months  E: Anamnesis of medical or drug abuse, surgery on the musculoskeletal system of the trunk, known congenital malformation of the spine or scoliosis, systemic-neurological-degenerative disease, history of stroke, psychiatric disorder, pregnancy and abnormal blood pressure | 39.0  (5.4) | 59 | 81.7  (15.7) | 177.5  (6.5) | 25.9  (4.7) | 11.6  (9.9) | TSK:  B – 23.8  (8.6)  ODI (%)  B – 21.1  (7.8) |
|  | Healthy | 20 | I: No LBP in the previous year or LBP lasting longer than one week in the previous 3 years, aged 31-50 years old  E: NR | 40.2  (5.4) | 62 | 77.5  (16.7) | 174.6  (8.9) | 25.2  (3.7) | N/A | N/A |
| Shahvarpour (2017);  Canada; Laboratory; Cohort Study | Pain | 33 | I: 18-65 years old adults who speak English or French and have lumbar or lumbosacral pain (with or without radicular pain) for at least 4 weeks (non-acute phase); and a score higher than 12% on the Oswestry Disability Index (ODI).  E: BMI > 30 kg/m^2^, prior surgery of the pelvis or spinal column, scoliosis, systemic or degenerative disease, a positive response to the Physical Activity Readiness Questionnaire, history of neurological diseases or deficits not related to back pain (e.g. Stroke, peripheral neuropathies and balance deficits), use of anticonvulsive, antidepressive and anxiolytic medication (use of antispasmodic, anti-inflammatory and analgesic drugs for back pain were accepted), pregnancy, claustrophobia, abnormal arterial blood pressure (hypertension). | M:  44.5  (13.9)  F:  47.8  (12.2) | 47.8 | M:  76.5  (13.2)  F:  71.5  (10.1) | M: 172.2  (6.4)  F:  163.1  (5.9) | M:  25.8  (3.9)  F:  26.8  (3.1) | NR | M:  ODI (%):  B – 26.4  (8.2)  NPRS (/10):  B – 5.0  (1.7)  F:  ODI (%):  B – 30.8  (10.2)  NPRS (/10):  B – 4.8  (1.0) |
|  | Healthy | 30 | I: 18-65 years old adults who speak English or French  E: Same as pain population | M:  39.3  (14.3)  F:  39.8  (14.1) | 50 | M:  77.1  (10.1)  F:  62.9  (10.6) | M:  178.1  (8.4)  F:  164.2  (5.9) | M:  24.4  (3.2)  F:  23.3  (3.6) | N/A | N/A |
| Watson (1997);  United Kingdom; Laboratory; Cross-sectional | Pain  (Test-retest) | 11 | I: Chronic low back pain as the primary presenting condition, duration of at least 6 months, not undergoing any current treatment other than routine analgesia, willingness to participate in a pain management program, aged between 18 and 65 years.  E: Major structural abnormality (e.g., kyphoscoliosis), evidence of inflammatory, systemic or neoplastic disease, major psychiatric illness, pregnancy, and any other medical condition likely to interfere with an active rehabilitation program. | 39.4  (6.9) | 55 | NR | NR | NR | 4.4  (4.1) | NR |
|  | Pain  (Validity) | 70 | I: Same as test-retest group  E: Same as test-retest group | 42.9  (9.2) | 49 | NR | NR | NR | 6.5  (6.1) | NR |
|  | Healthy | 20 | I: Never professionally consulted (therapist or doctor) at any time for low back pain, no episodes of low back pain/backache in the last 6 months, not on any current medication, no history of chronic musculoskeletal or abdominal pain, aged between 18 and 65 years.  E: NR | 38.6  (9.2) | 55 | NR | NR | NR | N/A | N/A |

A = Analysis, B = Baseline, BDI = Beck Depression Inventory, BPFS = Back Pain Functional Scale, E = Exclusion Criteria, F = Females, FLAG = Flexion Aggravating Questionnaire, I = Inclusion Criteria, IQR = Interquartile Range, MVADS = Million Visual Analog Disability Scale, N/A = Not Applicable, NDI = Neck Disability Index, NPRS = Numeric Pain Rating Scale, NR = Not Reported, ODI = Oswestry Disability Index, R-ODI = Revised Oswestry Disability Index, RMDQ = Roland Morris Disability Questionnaire, TSK = Tampa Scale of Kinesiophobia, VAS = Visual Analoge Scale, 95%CI = 95 Percent Confidence Interval.
