# Supplementary material for: A systematic review and meta analysis of measurement properties for the flexion relaxation ratio in people with and without non specific spine pain
Source: Sci Rep. 2024 Feb 8;14:3260. doi: 10.1038/s41598-024-52900-z (PMC10853169; doi:10.1038/s41598-024-52900-z)
Supplement: Supplementary file 4 — Supplementary Information 2. [file 41598_2024_52900_MOESM4_ESM.docx]

Supplementary Information 2 – Flexion relaxation ratio systematic review and meta-analysis search strategies.

All searches were run May 18, 2021 and updated (re-run) June 1, 2022.

**MEDLINE (via Ovid)**

Ovid MEDLINE(R) and Epub Ahead of Print, In-Process, In-Data-Review & Other Non-Indexed Citations and Daily <1946 to May 17, 2021>

| 1 | (flexion relaxation or fr response* or fr phenomen* or fr ratio* OR FRR OR FRP).tw,kf |
| --- | --- |
| 2 | exp Back/ |
| 3 | exp Back Muscles/ |
| 4 | exp Back Pain/ |
| 5 | exp Neck/ |
| 6 | Neck Muscles/ |
| 7 | Neck Pain/ |
| 8 | exp Spine/ |
| 9 | (back or trunk or lumbar or lumbosacral or lumbo sacral or lumbopelvic or lumbo pelvic or multifidus or longissimus or iliocostalis or erector spinae or quadratus lumborum or thoracic or neck or cervical or spine or spinal or paraspinal or vertebr*).tw,kf |
| 10 | (dorsalgi* or lumbalgi* or lumbago or lumbodyni* or backache* or neckache* or lbp or nsbp or nslbp or cnsbp or cnslbp).tw,kf |
| 11 | or/2-10 |
| 12 | 1 and 11 |
| 13 | Animals/ not Humans/ |
| 14 | 12 not 13 |

**Embase (via Embase.com)**

| #1 | 'flexion relaxation':ti,ab,kw OR 'fr response*':ti,ab,kw OR 'fr phenomen*':ti,ab,kw OR 'fr ratio*':ti,ab,kw OR frr:ti,ab,kw OR frp:ti,ab,kw |
| --- | --- |
| #2 | 'back'/exp |
| #3 | 'back muscle'/exp |
| #4 | 'backache'/exp |
| #5 | 'neck'/exp |
| #6 | 'neck muscle'/exp |
| #7 | 'neck pain'/exp |
| #8 | 'spine'/exp |
| #9 | 'spinal pain'/de |
| #10 | back:ti,ab,kw OR trunk:ti,ab,kw OR lumbar:ti,ab,kw OR lumbosacral:ti,ab,kw OR 'lumbo sacral':ti,ab,kw OR lumbopelvic:ti,ab,kw OR 'lumbo pelvic':ti,ab,kw OR multifidus:ti,ab,kw OR longissimus:ti,ab,kw OR iliocostalis:ti,ab,kw OR 'erector spinae':ti,ab,kw OR 'quadratus lumborum':ti,ab,kw OR thoracic:ti,ab,kw OR neck:ti,ab,kw OR cervical:ti,ab,kw OR spine:ti,ab,kw OR spinal:ti,ab,kw OR paraspinal:ti,ab,kw OR vertebr*:ti,ab,kw |
| #11 | dorsalgi*:ti,ab,kw OR lumbalgi*:ti,ab,kw OR lumbago:ti,ab,kw OR lumbodyni*:ti,ab,kw OR backache*:ti,ab,kw OR neckache*:ti,ab,kw OR lbp:ti,ab,kw OR nsbp:ti,ab,kw OR nslbp:ti,ab,kw OR cnsbp:ti,ab,kw OR cnslbp:ti,ab,kw |
| #12 | #2 OR #3 OR #4 OR #5 OR #6 OR #7 OR #8 OR #9 OR #10 OR #11 |
| #13 | #1 AND #12 |
| #14 | [animals]/lim NOT [humans]/lim |
| #15 | #13 NOT #14 |

**CINAHL Plus (via EBSCOhost)**

| S1 | TI ("flexion relaxation" OR "FR response*" OR "FR phenomen*" OR "FR ratio*" OR FRR OR FRP) OR AB ("flexion relaxation" OR "FR response*" OR "FR phenomen*" OR "FR ratio*" OR FRR OR FRP) |
| --- | --- |
| S2 | (MH "Back") |
| S3 | (MH "Erector Spinae Muscles") OR (MH "Latissimus Dorsi Muscles") OR (MH "Multifidus Muscles") OR (MH "Quadratus Lumborum Muscles") |
| S4 | (MH "Back Pain+") |
| S5 | (MH "Neck+") |
| S6 | (MH "Neck Muscles+") |
| S7 | (MH "Neck Pain") |
| S8 | (MH "Spine+") |
| S9 | TI (back OR trunk OR lumbar OR lumbosacral OR "lumbo sacral" OR lumbopelvic OR "lumbo pelvic" OR multifidus OR longissimus OR iliocostalis OR "erector spinae" OR "quadratus lumborum" OR thoracic OR neck OR cervical OR spine OR spinal OR paraspinal OR vertebr*) OR AB (back OR trunk OR lumbar OR lumbosacral OR "lumbo sacral" OR lumbopelvic OR "lumbo pelvic" OR multifidus OR longissimus OR iliocostalis OR "erector spinae" OR "quadratus lumborum" OR thoracic OR neck OR cervical OR spine OR spinal OR paraspinal OR vertebr*) |
| S10 | TI (dorsalgi* OR lumbalgi* OR lumbago OR lumbodyni* OR backache* OR neckache* OR lbp OR nsbp OR nslbp OR cnsbp OR cnslbp) OR AB (dorsalgi* OR lumbalgi* OR lumbago OR lumbodyni* OR backache* OR neckache* OR lbp OR nsbp OR nslbp OR cnsbp OR cnslbp) |
| S11 | S2 OR S3 OR S4 OR S5 OR S6 OR S7 OR S8 OR S9 OR S10 |
| S12 | S1 AND S11 |

**SPORTDiscus (via EBSCOhost)**

| S1 | TI ("flexion relaxation" OR "FR response*" OR "FR phenomen*" OR "FR ratio*" OR FRR OR FRP) OR AB ("flexion relaxation" OR "FR response*" OR "FR phenomen*" OR "FR ratio*" OR FRR OR FRP) OR KW ("flexion relaxation" OR "FR response*" OR "FR phenomen*" OR "FR ratio*" OR FRR OR FRP) |
| --- | --- |
| S2 | DE "BACK" OR DE "BACK physiology" OR DE "LUMBOSACRAL region" OR DE "SACROCOCCYGEAL region" |
| S3 | DE "BACK muscles" OR DE "SPLENIUS muscles" OR DE "TRAPEZIUS muscle" OR DE "ERECTOR spinae muscles" |
| S4 | DE "BACKACHE" OR DE "LUMBAR pain" |
| S5 | DE "NECK" OR DE "NECK physiology" OR DE "CERVICAL vertebrae" |
| S6 | DE "NECK muscles" |
| S7 | DE "NECK pain" |
| S8 | DE "SPINE" OR DE "SPINE physiology" OR DE "VERTEBRAE" |
| S9 | TI (back OR trunk OR lumbar OR lumbosacral OR "lumbo sacral" OR lumbopelvic OR "lumbo pelvic" OR multifidus OR longissimus OR iliocostalis OR "erector spinae" OR "quadratus lumborum" OR thoracic OR neck OR cervical OR spine OR spinal OR paraspinal OR vertebr*) OR AB (back OR trunk OR lumbar OR lumbosacral OR "lumbo sacral" OR lumbopelvic OR "lumbo pelvic" OR multifidus OR longissimus OR iliocostalis OR "erector spinae" OR "quadratus lumborum" OR thoracic OR neck OR cervical OR spine OR spinal OR paraspinal OR vertebr*) OR KW (back OR trunk OR lumbar OR lumbosacral OR "lumbo sacral" OR lumbopelvic OR "lumbo pelvic" OR multifidus OR longissimus OR iliocostalis OR "erector spinae" OR "quadratus lumborum" OR thoracic OR neck OR cervical OR spine OR spinal OR paraspinal OR vertebr*) |
| S10 | TI (dorsalgi* OR lumbalgi* OR lumbago OR lumbodyni* OR backache* OR neckache* OR lbp OR nsbp OR nslbp OR cnsbp OR cnslbp) OR AB (dorsalgi* OR lumbalgi* OR lumbago OR lumbodyni* OR backache* OR neckache* OR lbp OR nsbp OR nslbp OR cnsbp OR cnslbp) OR KW (dorsalgi* OR lumbalgi* OR lumbago OR lumbodyni* OR backache* OR neckache* OR lbp OR nsbp OR nslbp OR cnsbp OR cnslbp) |
| S11 | S2 OR S3 OR S4 OR S5 OR S6 OR S7 OR S8 OR S9 OR S10 |
| S12 | S1 AND S11 |

**Web of Science Core Collection**

Editions Searched:

- Science Citation Index Expanded (SCI-EXPANDED) --1900-present
- Social Sciences Citation Index (SSCI) --1956-present
- Arts & Humanities Citation Index (A&HCI) --1975-present
- Conference Proceedings Citation Index- Science (CPCI-S) --1990-present
- Conference Proceedings Citation Index- Social Science & Humanities (CPCI-SSH) --1990-present
- Emerging Sources Citation Index (ESCI) --2015-present

| # 1 | TS=("flexion relaxation" OR "FR response*" OR "FR phenomen*" OR "FR ratio*" OR FRR OR FRP) |
| --- | --- |
| # 2 | TS=(back OR trunk OR lumbar OR lumbosacral OR "lumbo sacral" OR lumbopelvic OR "lumbo pelvic" OR multifidus OR longissimus OR iliocostalis OR "erector spinae" OR "quadratus lumborum" OR thoracic OR neck OR cervical OR spine OR spinal OR paraspinal OR vertebr*) |
| # 3 | TS=(dorsalgi* OR lumbalgi* OR lumbago OR lumbodyni* OR backache* OR neckache* OR lbp OR nsbp OR nslbp OR cnsbp OR cnslbp) |
| #4 | #3 OR #2 |
| # 5 | #4 AND #1 |
| # 6 | TS=(((fiber* OR fibre* OR fabric) NEAR/0 reinforce* NEAR/0 (polymer* OR plastic*)) OR "fire radiative power") |
| # 7 | #5 NOT #6 |

**Scopus**

| 1 | TITLE-ABS-KEY ("flexion relaxation" OR "FR response*" OR "FR phenomen*" OR "FR ratio*" OR frr OR frp) |
| --- | --- |
| 2 | TITLE-ABS-KEY (back OR trunk OR lumbar OR lumbosacral OR "lumbo sacral" OR lumbopelvic OR "lumbo pelvic" OR multifidus OR longissimus OR iliocostalis OR "erector spinae" OR "quadratus lumborum" OR thoracic OR neck OR cervical OR spine OR spinal OR paraspinal OR vertebr*) |
| 3 | TITLE-ABS-KEY (dorsalgi* OR lumbalgi* OR lumbago OR lumbodyni* OR backache* OR neckache* OR lbp OR nsbp OR nslbp OR cnsbp OR cnslbp) |
| 4 | #3 OR #4 |
| 5 | #1 AND #4 |
| 6 | TITLE-ABS-KEY (((fiber* OR fibre* OR fabric) PRE/0 reinforce* PRE/0 (polymer* OR plastic*)) OR "fire radiative power") |
| 7 | #5 NOT #6 |
