# Supplementary material for: A systematic review and meta analysis of measurement properties for the flexion relaxation ratio in people with and without non specific spine pain
Source: Sci Rep. 2024 Feb 8;14:3260. doi: 10.1038/s41598-024-52900-z (PMC10853169; doi:10.1038/s41598-024-52900-z)
Supplement: Supplementary file 5 — Supplementary File 3a. [file 41598_2024_52900_MOESM5_ESM.docx]

Supplementary File 3a - Summary of Results for Responsiveness

We did not find any of the studies were adequately set up to test the measurement property of responsiveness for the cervical, thoracic, or lumbar spine. We have included the characteristics, risk of bias ratings, and a summary of exposures tested in the supplementary files as the information may be helpful to researchers pursuing work in this area.

*Risk of Bias*

For responsiveness of the cervical FRR, most studies received a doubtful overall score [64–67,69,72,75], one received inadequate [71], with the rest receiving overall scores of adequate or very good [42,60,68,70,73]. Similarly, for responsiveness of the lumbar FRR, most studies received an overall rating of doubtful [30,37,44,45,48,49,51,53,56–58,62,63], and the rest received an overall score of adequate [28,36,46,47,52,54,55,61].

*Summary of Exposures used with the assumption of responsiveness*

Studies compared the cervical FRR before and after exposure to a physical task (sitting [64], static end range flexion [67], smart phone posture [73], overhead work [72], computer work [71], below knee assembly work [60], assembly work [74], therapeutic intervention (exercise [66,68], spinal manipulation [68], stretching [70], and fatigue protocols [42,69,75] (Table S4.1).

Studies compared the lumbar FRR before and after exposure to a physical task (standing [37], below knee assembly [60]), therapeutic interventions (lumbar support [45], spinal manipulation [46,53,57,63], Kinesiotape [48], physical therapy [30,50,57], exercise [50–53,55,58], and stretching [54] (Table S4.2).
