# Supplementary material for: A systematic review and meta analysis of measurement properties for the flexion relaxation ratio in people with and without non specific spine pain
Source: Sci Rep. 2024 Feb 8;14:3260. doi: 10.1038/s41598-024-52900-z (PMC10853169; doi:10.1038/s41598-024-52900-z)
Supplement: Supplementary file 6 — Supplementary Table 3b. [file 41598_2024_52900_MOESM6_ESM.docx]

Supplementary Table 3b – Characteristics of included studies of thoracic and cervical flexion relaxation ratio (FRR) responsiveness. Mean (Standard Deviation) given unless otherwise stated. If Baseline and Analysis n are the same than only one value is included. * Indicates a standard deviation of height as reported in the retrieved article that we believe is incorrect.

| **Author (Year); Country; Setting; Design** | **Group** | **n** | **(I)nclusion, (E)xclusion Criteria** | **Age**  **(yr)** | **%F** | **Mass**  **(kg)** | **Height**  **(cm or m)** | **BMI**  **(kg/m^2^)** | **Duration**  **(mos or yr)** | **Outcome** |
| --- | --- | --- | --- | --- | --- | --- | --- | --- | --- | --- |
| *Thoracic* | | | | | | | | | | |
| Yoo (2016);  Korea; Laboratory; Cross-sectional | Healthy | 10 | I: No history of musculoskeletal disorders or pain associated with the upper extremity or spine in the past 6 months  E: NR | 20-30 | 0 | 69.1  (5.9) | 172.5  (3.2) | NR | N/A | N/A |
| *Cervical* | | | | | | | | | | |
| Choi (2020);  South Korea; Laboratory; Cross-sectional | Pain  (Low FRR) | 7 | I: Graduate students who worked on computers for > 4 hours/day. FRR ≤ 2.5  E: History of pain/MSD in neck, shoulder, upper limbs and back | 27.6  (3.1) | 0 | 81.4  (14.3) | 172.9  (2.9) | NR | NR | FRR:  B – 2.0  (0.5) |
|  | Pain  (High FRR) | 16 | I: Same as Low FRR group, except FRR > 2.5  E: Same as Low FRR group | 26.4  (2.5) | 0 | 74.6  (7.7) | 174.2  (3.8) | NR | NR | FRR:  B – 3.9  (1.4) |
| Ding (2020);  China; Hospital; Cohort | Pain  (Knee flexion contracture) | 22 | I: Hospital inpatient in department of orthopedic surgery, age 18-70 years old, BMI ≤ 31, knee flexion contracture ≥ 5 degrees  E: Spinal deformity, spinal tumor, compression fracture, degenerative spinal diseases that can affect spinal alignment, history of spine surgery, history of knee surgery, hip joint disorder, rheumatoid arthritis, ankylosing spondylitis, Parkinson’s disease, other nerve system diseases | 61.9  (8.9) | 73 | NR | NR | 24.9  (2.3) | 12.5 mos knee pain (14.3) | NDI (%):  B – 19.27  (10.4)  F/U – 9.18  (5.84)  VAS (cm):  B – 2.64  (1.23)  F/U – 1.05  (0.96) |
|  | Pain  (Surgical inpatient) | 12 | I: Hospital inpatient who had surgery to remove internal fixation from upper limbs. Same criteria for age, sex and BMI as KFC group  E: Same as Knee Flexion Contracture group | 57.4  (10.1) | 75 | NR | NR | 25.4  (2.1) | NR | NDI (%):  B – 2.83  (1.99)  VAS (cm):  B – 0.75  (0.75) |
| Hyun-Mu (2016);  South Korea; Laboratory; Cohort | Pain | 15 | I: College students, diagnosis of simple video display terminal syndrome, right-handed  E: History of neck and spinal damage, undergone surgery to the neck bones, unable to conduct study’s training program | 28.53  (4.44) | NR | 59.53  (11.70) | 168.47  (8.00) | NR | NR | VAS (/10):  B – 3.67  (1.40)  F/U – 1.73  (1.10) |
| Mousavi-Khatir (2016); Iran; Laboratory; Cross-sectional | Healthy | 40 | I: Between 20-35 years of age, with no reported previous episodes of spinal disorder or any self-reported history of neck, shoulder, back pain or current medical conditions. Physical examination by an experienced physiotherapist to ensure the health of the musculoskeletal system  E: NR | 22.6  (3.1) | 50 | 65.1  (11) | 1.70  (0.8)* | 22.3  (2.5) | N/A | N/A |
| Murphy (2010a);  Canada; Laboratory; RCT |  | B: 20 |  |  |  |  |  |  |  |  |
|  | Pain  (Manipulation and exercise | A: 8 | I: People with chronic non-specific neck pain, defined as pain that persists for more than 3 months, who were free from mechanical neck injuries and had no significant pathology (assessed by their general practitioner). Free of neurologic or cardiovascular disease that would contraindicate exercise as a safe intervention, based on American College of Sports Medicine guidelines  E: Received cervical manipulation or exercises in the last 3 months and if they showed any contraindications to cervical spine manipulation such as spinal instability, recurrent dizziness or vertigo, hypertension, or upper limb radiculopathy | 43  (8) | 75 | NR | NR | 25  (4) | 9  (9) | NDI (/50):  B – 25  (8.8)  F/U – 14.3  (11.3)  VAS_current_ (mm):  B – 33.6  (22.12)  F/U – 16.9  (12.54)  VAS_worst_ (mm):  B – 51.5  (21.06)  F/U – 42  (19.48) |
|  | Pain  (Exercise only) | A: 7 | I: Same as Manipulation and exercise group  E: Same as Manipulation and exercise group | 44  (10) | 71 | NR | NR | 28  (5) | 7  (5) | NDI (/50):  B – 24.9  (7.9)  F/U – 16.6  (9.5)  VAS_current_ (mm):  B – 33.0  (18.10)  F/U – 20.29  (13.54)  VAS_worst_ (mm):  B – 51.71  (24.34)  F/U – 31.86  (17.44) |
| Nimbarte (2014a);  USA; Laboratory; Cross-sectional | Healthy | 13 | I: Healthy male participants, free of any type of musculoskeletal disorders and no history of neck and/or shoulder injury or notable neck pain that required medical care over the last 12 months  E: Cardiac and other health issues (chest pain, dizziness, and heart problems), screened with the PAR-Q | 29.8  (7.3) | 0 | 72.8  (12.2) | 172.8  (5.4) | NR | N/A | N/A |
| Nimbarte (2014b); USA; Laboratory; Cross-sectional | Healthy | 20 | I: Free from any type of musculoskeletal, degenerative, or neurological disorders and had no history of neck, shoulder, and back pain or notable pain that required medical care over the last 12 months  E: NR | F:  29.1  (3.9)  M:  24.1  (2.4) | 50 | F:  56.2  (7.7)  M:  70.5  (7.6) | F:  162.9  (6.3)  M:  175.6  (6.3) | NR | N/A | N/A |
| Park (2019);  Korea; Laboratory; RCT | Pain  (Static stretching) | 12 | I: Sedentary workers who had neck discomfort or pain in daily life, regularly performed seated work >4 hours/day  E: History of upper or lower extremity injuries that affected daily life, other than neck pain and discomfort | 21.75  (0.97) | NR | 61.58  (7.46) | 166.17  (5.20) | 22.05  (2.06) | NR | NDI (/50):  B – 3.75  (1.48) |
|  | Pain  (Dynamic stretching) | 12 | I: Same as Static stretching group  E: Same as Static stretching group | 21.67  (0.89) | NR | 63.92  (17.35) | 166.83  (9.42) | 22.64  (3.67) | NR | NDI (/50):  B – 5.33  (2.10) |
| Shin (2014c); Korea; Laboratory; Cross-sectional | Healthy | 15 | I: Healthy  E: Experience of neck pain, spinal trauma or cervical surgery in the past year | 26.07  (5.73) | NR | 69.33  (13.01) | 173.2  (10.05) | NR | N/A | VAS:  B – 0  F/U_desk_ – 1.7  F/U_lap_ – 5.2 |
| Shin (2012); Korea; Laboratory; Cross-Sectional | Healthy | 14 | I: NR  E: No neck and/or back pain for a minimum of 1 year before the study. No upper limb or cervical spine pathology, no rheumatological or neurological conditions | 26.3  (3.2) | 43 | 61.5  (9.7) | 168.7  (6.9) | NR | N/A | N/A |
| Shin (2014b); Korea; Laboratory; Cross-sectional | Healthy | 12 | I: Healthy and free of neck and back pain for a minimum of 1 year before the study  E: Upper-limb or cervical spine pathologies, rheumatological or neurological conditions | 25.0  (3.5) | 75 | 57.0  (9.79) | 165.0  (7.66) | NR | N/A | N/A |
| Shin (2014a); Korea; Laboratory; Cross-sectional | Pain | 15 | I: NR  E: Current back or neck pain or with past lower back pain, cervical or thoracic pain, spinal trauma or surgery | 21.2  (1.3) | 0 | 65.7  (9.6) | 174.1  (6.0) | NR | N/A | VAS_neck_:  B – 15.0  (17.3)  VAS_back_:  B – 16.5  (19.6) |
| Yoo (2014);  Korea;  Laboratory; Cross-sectional | Healthy | 15 | I: Healthy and free of any neck and back pain for min 1 year before the study  E: Upper limb or cervical spine pathologies, rheumatological or neurological conditions | 21-32 | 0 | 65.6  (5.7) | 176.8  (3.8) | NR | N/A | N/A |
| Zabihhosseinian (2015);  Canada; Laboratory; Cross-sectional | Pain | B: 12  A: 11 | I: Mild (NDI scores of 5-14) to moderate (NDI scores of 15-24) neck pain for at least 3 months  E: NR | 23.1  (3.81) | 58 | 73.1  (21.57) | 168.75  (15.38) | NR | 3  (2) | Repetition of neck pain (#/wk):  B – 4  (2)  NDI:  B – 9.75  (3.88) |
|  | Healthy | B: 13  A: 11 | I: Free from chronic or recurrent neck, shoulder or elbow pain for at least 3 months prior (NDI scores of 0-4)  E: NR | 25.76  (4.51) | 46 | 66.15  (13.41) | 168.69  (8.91) | NR | N/A | N/A |

A = Analysis, B = Baseline, BDI = Beck Depression Inventory, E = Exclusion Criteria, F = Females, F/U = Follow up, FLAG = Flexion Aggravating Questionnaire, FRR = Flexion Relaxation Ratio, I = Inclusion Criteria, IQR = Interquartile Range, MVADS = Million Visual Analog Disability Scale, N/A = Not Applicable, NDI = Neck Disability Index, NPRS = Numeric Pain Rating Scale, NR = Not Reported, ODI = Oswestry Disability Index, R-ODI = Revised Oswestry Disability Index, RMDQ = Roland Morris Disability Questionnaire, TSK = Tampa Scale of Kinesiophobia, VAS = Visual Analoge Scale, 95%CI = 95 Percent Confidence Interval.
