# Supplementary material for: A systematic review and meta analysis of measurement properties for the flexion relaxation ratio in people with and without non specific spine pain
Source: Sci Rep. 2024 Feb 8;14:3260. doi: 10.1038/s41598-024-52900-z (PMC10853169; doi:10.1038/s41598-024-52900-z)
Supplement: Supplementary file 7 — Supplementary Table 3c. [file 41598_2024_52900_MOESM7_ESM.docx]

Supplementary Table 3c – Characteristics of included studies of lumbar flexion relaxation ratio (FRR) responsiveness. Mean (Standard Deviation) given unless otherwise stated. If Baseline and Analysis n are the same than only one value is included. * Indicates a standard deviation of height as reported in the retrieved article that we believe is incorrect.

| **Author (Year); Country; Setting; Design** | **Group** | **n** | **(I)nclusion, (E)xclusion Criteria** | **Age**  **(yr)** | **%F** | **Mass**  **(kg)** | **Height**  **(cm, m)** | **BMI**  **(kg/m^2^)** | **Duration**  **(mos, yr)** | **Outcome** |
| --- | --- | --- | --- | --- | --- | --- | --- | --- | --- | --- |
| Shin (2014); Korea; Laboratory; Cross-sectional | Healthy | 15 | I: NR  E: Current back or neck pain or with past lower back pain, cervical or thoracic pain, spinal trauma or surgery. | 21.2  (1.3) | 0 | 65.7  (9.6) | 174.1  (6.0) | NR | N/A | VAS_neck_:  B – 15.0  (17.3)  VAS_back_:  B – 16.5  (19.6) |
| Arguisuelas (2019);  Spain; Laboratory; RCT | Pain  (MFR) | B: 18  A: 12 | I: 18-60 years; diagnosis of non-specific CLBP of at least 3 months duration  E: Pregnant or met any of the  following criteria: suffering from a spinal tumor, infection, or fracture, autoimmune, infectious, vascular, endocrine, metabolic, or neoplastic systemic disease, fibromyalgia, cauda equina syndrome, submission to a previous spine surgery, or musculoskeletal injuries of the lower limbs. Other exclusion criteria were any of the contraindications described for myofascial treatment, previous experience with myofascial therapy, or a history of rehabilitation treatment for back pain within the preceding two months. | 47.2  (9.8) | 67 | NR | NR | 25.8  (4.8) | 6.8  (4.6) | SF-MPQ:  B – 21.5  (7.8)  RMDQ:  B – 8.8  (4.7) |
|  | Pain  (Sham MFR) | B: 18  A: 12 | I: Same as MFR group  E: Same as MFR group | 48.6  (10.1) | 67 | NR | NR | 25.8  (3.7) | 8  (8.2) | SF-MPQ:  B – 22.2  (9.9)  RMDQ:  B – 11  (4.6) |
| Bataller-Cervero (2019);  Spain; Laboratory; RCT | Pain  (Lumbar support while working) | 14 | I: Line workers at electrical appliances manufacturing, between 35-50 years old, sick leave related to lumbar pain in previous 2 years, working in a standing posture on an assembly line  E: Short-term contract with company, lower than “90% punctuation” in lumbar functionality test | 43  (7) | 21.5 | 79.1  (13.5) | 172  (10.0) | 26.4  (2.8) | NR | NPRS (/5):  B – 3.6  (0.8) |
|  | Pain (No intervention control) | 14 | I: Same as Intervention group  E: Same as Intervention group | 41  (7) | 21.5 | 82.2  (20.0) | 173  (10.1) | 23.3  (6.1) | NR | NPRS (/5):  B – 2.7  (1.7) |
| Bicalho (2010);  Brazil; Laboratory; RCT | Pain  (Spinal manipulation) | 20 | I: 18-55 years old, absence of back pain for ≥ 3 months, no treatment or spinal manipulation in last 6 months  E: Pain radiating below knee, skeletal or neuromuscular disorders identified on imaging (MRI, radiograph), Accident Compensation Corporation red flags | 29.5  (9.64) | 75 | 73.05  (18.04) | 1.67  (0.08) | 24.25  (2.75) | 5.26  (4.97) | ODI (%):  B – 14.6  (5.62)  VAS (/100):  B – 38.80  (23.08) |
|  | Pain (No intervention control) | 20 | I: Same as Intervention group  E: Same as Intervention group | 26.5  (8.27) | 60 | 73.05  (18.04) | 1.72  (0.10) | 24.34  (3.96) | 3.48  (3.35) | ODI (%):  B – 16.60  (7.37)  VAS (/100):  B – 38.40  (17.80) |
| Descarreaux  (2008); Canada; Laboratory; RCT  (Crossover) | Healthy | 20 | I: No history of low back pain  E: Present or past low back or thoracic pain, spinal trauma or surgery | 23.9  (4.0) | 55 | 64.1  (13.1) | 1.70  (0.10) | 22.2  (3.2) | N/A | N/A |
| Grzeskowiak (2019);  Poland; Laboratory; RCT | Pain  (Kinesiotape) | B: 20  A: 19 | I: 20-55 years old, MRI confirmed unilateral or central disc herniation at L4/L5 and/or L5/S1, RMDQ ≥ 4, lumbar or lumbosacral pain with or without referring to leg for at least 3 months  E: Disc degeneration or herniation(s) to levels other than L4/L5 and L5/S1, coexisting systemic or orthopedic diseases, pregnancy, coexisting pathologies of spinal column and pelvis, previous kinesiotape therapy, no physical therapy referral at study time, BMI > 30 | 36.5  (8.9) | 68 | NR | NR | 24.7  (2.8) | NR | RAPA-A (/7):  B – 3.7  (1.9)  RAPA-SF (/3):  B – 0.9  (1.1)  RMDQ:  B – 8.7  (3.9)  Pain_actual_:  B – 3.3  (2.1)  Pain_average_:  B – 4.9  (1.7)  Pain_best_:  B – 1.5  (0.9)  Pain_worst_:  B – 8.5  (1.1) |
|  | Pain  (Placebo taping) | B: 20  A: 19 | I: Same as Intervention group  E: Same as Intervention group | 36.4  (10.5) | 79 | NR | NR | 24.4  (3.3) | NR | RAPA-A (/7):  B – 3.5  (1.4)  RAPA-SF (/3):  B – 1.2  (0.9)  RMDQ:  B – 7.3  (3.7)  Pain­_actual_:  B – 3.3  (1.9)  Pain_average_:  B – 5.3  (1.8)  Pain_best_:  B – 1.8  (1.2)  Pain_worst_:  B – 7.9  (1.8) |
| Horn (2013);  USA; Laboratory; Cohort | Healthy (Analysed sample divided in half based on post-exercise pain) | B: 51  A: 42 | I: NR  E: Previous participation in trunk extensor conditioning program, current low back pain, chronic medical condition that might affect pain perception, kidney dysfunction, muscle damage, major psychiatric disorder, prior injury including surgery to the lumbar spine, renal malfunction, cardiac condition, high blood pressure, osteoporosis, liver dysfunction, intervention for symptoms induced by exercise before termination of study | 22.1  (3.5) | 61 | NR | NR | 23.9  (4.5) | N/A | N/A |
| Kim (2013);  South Korea; Laboratory; RCT | Pain (Neurac sling lumbar stabilization exercise) | 8 | I: Pain duration > 12 weeks, pain localized between L2-L4 and the inferior gluteal folds, no surgery to lumbar area due to orthopaedic problems, VAS and ODI ≥ 6, no severe modification or fracture based on x-ray, no sensory nervous system disorder, no vestibular system disorder, no nervous or respiratory system disease  E: NR | 46.6  (19.7) | 50 | 61.3  (10.4) | 165.7  (4.0) | 22.3  (3.3) | 12.7 mos  (3.0) | VAS:  B – 6.9  (0.7)  F/U – 3.3  (1.1)  ODI:  B – 20.0  (4.0)  F/U – 7.4  (2.1) |
|  | Pain  (Ordinary physical exercise) | 8 | I: Same as Intervention group  E: Same as Intervention group | 48.6  (9.9) | 63 | 57.9  (4.9) | 164.6  (4.8) | 21.3  (0.8) | 18.7*  (12.2) | VAS:  B – 7.0  (0.8)  F/U – 2.3  (1.3)  ODI:  B – 20.4  (3.9)  F/U – 6.0  (2.4) |
| Lalanne (2009);  Canada; Laboratory; Randomized Control Trial | Pain  (Spinal manipulation) | 13 | I: Age 18-60 y/o, chronic low back pain (constant or recurrent) > 6 mos  E: Spondylolisthesis, axial skeletal inflammation or osteoarthritis, collagenosis, osteoporosis, spinal surgery, neuromuscular disease, lower limb musculoskeletal injuries, malignant tumor, hypertension, infection or any other nonmechanical condition, radiculopathy, progressive neurological deficit, myelopathy, herniated lumbar disk, severe pain (> 7 on VAS) | 36.1  (12.3) | 38 | 77.1  (17.4) | 1.73  (0.12) | 25.6  (3.7) | <1: 1  1-3: 4  3-5: 3  5-10: 2  >10: 3 | mODI:  B – 19.2  (10.1)  FABQ-W (/42):  B – 16.1  (9.8)  FABQ-PA (/24):  B – 8.1  (4.4)  VAS (/100):  B – 26.9  (21.8)  F/U – 24.9  (22.3) |
|  | Pain  (No intervention control) | 14 | I: Same as Intervention group  E: Same as Intervention group | 43.5  (10.5) | 57 | 72.4  (16.5) | 1.68  (0.10) | 25.3  (3.8) | <1: 2  1-3: 2  3-5: 3  5-10: 4  >10: 3 | mODI:  B – 15.6  (8.9)  FABQ-W (/42):  B – 11.9  (8.5)  FABQ-PA (/24):  B – 7.6  (6.3)  VAS (/100):  B – 23.3  (21.8)  F/U – 30.1  (26.9) |
| Mak (2010);  Hong Kong; Laboratory; Cohort | Pain | 25 | I: Back pain > 3 months  E: Prior back surgery, clinically identifiable pathology (e.g., herniated disc), nerve root compression, spinal stenosis, spondylolisthesis, inflammatory arthritis, cancer, pregnant, medical condition that could be exacerbated by participation | 42.2  (10.56) | 12 | NR | NR | 24.0  (5.6) | NR | ODI:  B – 46.64  (10.09)  F/U – 41.58  (13.51)  VAS_rest_ (/100):  B – 31.16  (18.50)  F/U – 31.56  (18.76)  VAS_exertion_:  B – 68.88  (15.46)  F/U – 66.96  (18.53) |
|  | Healthy | 20 | I: No history of significant back pain  E: NR | 29.2  (4.75) | 15 | NR | NR | 25.3  (2.6) | N/A | N/A |
| Marshall (2006a);  New Zealand; Laboratory; Cohort | Pain | B: 20  A: 18 | I: 18-65 y/o, back pain for at least 12 weeks, had not received specific abdominal stabilization training or spinal manipulation, not performed an organized regimen of Swiss ball training in previous 3 mos  E: Severe postural or skeletal abnormalities, obvious neuromuscular disorder, spinal damage identified by MRI or radiograph, any Accident Compensation Corporation red flags | 38.8  (12.1) | 40 | 76.15  (7.21) | 1.76  (0.06) | NR | 4.8 | VAS (%):  B – 47.39  (23.72)  F/U_12w_ – 23.49  (19.71)  F/U_3mo_ – 20  (15.82)  SF-12_physical_:  B – 44.15  (6.86)  F/U_12w_ – 48.25  (7.46)  F/U_3mo_ – 48.78  (7.29)  SF-12_psych_:  B – 48.45  (9.22)  F/U_12w_ – 55.77  (3.77)  F/U_3mo_ – 51.59  (6.7)  SEES (%):  B – 90.64  (8.79)  F/U_12w_ – 85.69  (18.88)  F/U_3mo_ – 81.08  (16.85)  ODI (%):  B – 24.0  (7.0)  F/U_12w_ –  F/U_3mo_ – |
| Marshall  (2008);  New Zealand; Laboratory; RCT |  | B: 60 |  |  |  |  |  |  |  |  |
|  | Pain (Spinal manipulation, Control exercise) | A: 13 | I: Chronic nonspecific low back pain for at least 3 mos.  E: Severe postural abnormality or neuromuscular disorder, previous diagnosis of pathology, confirmed by MRI or radiograph, that would contraindicate exercise or spinal manipulation, manipulative treatment in last 3 mos, previous participation in specific abdominal | 35.8  (10.4) | 54 | 73.6  (11.9) | 173  (4) | 24.6  (3.6) | 4.0  (2.0) | ODI (%):  B – 23.8 |
|  | Pain  (Spinal manipulation, Swiss ball exercise) | A: 12 | I: Same as Spinal manipulation, Control exercise group  E: Same as Spinal manipulation, Control exercise group | 34.3  (9.2) | 50 | 78.7  (14.8) | 173  (10) | 26.2  (2.9) | 3.0  (2.5) | ODI (%):  B – 24.3 |
|  | Pain  (Control exercise) | A: 13 | I: Same as Spinal manipulation, Control exercise group  E: Same as Spinal manipulation, Control exercise group | 41.7  (10.7) | 42 | 83.8  (13.6) | 174  (4) | 27.5  (4.1) | 2.7  (1.3) | ODI (%):  B – 28.0 |
|  | Pain  (Swiss ball exercise) | A: 12 | I: Same as Spinal manipulation, Control exercise group  E: Same as Spinal manipulation, Control exercise group | 33.9  (9.6) | 50 | 77.5  (11.8) | 173  (11) | 26.4  (2.5) | 5.0  (2.5) | ODI (%):  B – 25.9 |
| Moore (2015);  New Zealand; Laboratory; Prospective Cohort | Pain | 9 | I: Participants with chronic low back pain from the general public. Aged between 16 and 65 years old, had experienced chronic LBP for a minimum of 3 months, displayed an impaired FR (as identified at initial consultation, visually determined by observation of "more than usual" activity occurring during terminal flexion)  E: History of muscular spinal pathology, class-two obesity, or were involved in another physical rehabilitation intervention | 48.4  (12.7) | 77.7 | NR | NR | 27  (5.3) | 9.4  (7.8) | ODI:  B – 20.4  (9.2)  F – 12  (7.7)  Sit and Reach (cm):  B – 19.2  (13.9)  F – 24.6  (11.7)  NPRS:  B – 4.7  (1.3)  F – 2.9  (2.3) |
| Pagé (2015);  Canada; Laboratory; Cohort | Pain | 21 | I: Aged 18-60 years old with non-specific chronic LBP (defined as episodic or constant pain present for more than 12 weeks, located between the 12^th^ rib and the inferior gluteal fold for which no specific source of pain could be identified)  E: LBP of specific origin, spine surgery or trauma, scoliosis, neurologic disease, uncontrolled hypertension, pregnancy, incapacity to perform a trunk flexion, recent lumbar cortisone injection and being under medications known to impair physical effort and pain perception | 36.52  (11.81) | 38 | NR | NR | 22.55  (3.86) | 7.36  (5.44) | Episodic (n):  B – 10  Constant (n):  B – 11  VAS_1-week_ (%):  B – 24.62  (16.17)  VAS_current_ (%):  B – 18.24  (15.70)  ODI (%):  B – 13.40  (8.76)  TSK:  B – 33.05  (7.18) |
| Pool-Goudzwaard et al (2018); Netherlands; Laboratory; Cross-Sectional | Pain | 16 | I: Age between 20-60 years old, being able to read and understand Dutch  E: Specific LBP due to malignant processes and systematic disease as well as the inability to bend forward | 37  (11.9) | 50 | NR | NR | NR | NR | RMDQ:  B – 5.2  (4)  NPRS:  B – 4  (2.5) |
|  | Healthy  (Study 1) | 24 | I: Age between 20-60 years old, being able to read and understand Dutch  E: NR | 34  (12.3) | 46 | NR | NR | NR | N/A | N/A |
|  | Healthy  (Study 2) | 6 | I: Same as Study 1 group  E: Same as Study 1 group | 27  (11.9) | 67 | NR | NR | NR | N/A | N/A |
| Pouretezad (2018); Iran;  Laboratory; Cross-sectional | Healthy | 22 | I: Aged 18-40 years  E: History of LBP or leg pain over the past 1 year, auditory or cognitive (memory) deficit, and any rheumatologic or neurologic disorder | 25.68  (6.04) | 18 | 69.18  (8.86) | 172.31  (8.03) | NR | N/A | N/A |
| Ringheim (2015);  Norway; Laboratory; Cross-sectional | Pain | 17 | I: Diagnosed with chronic LBP for more than 3 months  E: Anamnesis of medical or drug abuse, surgery on the musculoskeletal system of the trunk, known congenital malformation of the spine or scoliosis, systemic-neurological-degenerative disease, history of stroke, psychiatric disorder, pregnancy and abnormal blood pressure | 39.0  (5.4) | 59 | 81.7  (15.7) | 177.5  (6.5) | 25.9  (4.7) | 11.6  (9.9) | TSK:  B – 23.8  (8.6)  ODI:  B – 21.1  (7.8) |
|  | Healthy | 20 | I: No LBP in the previous year or LBP lasting longer than one week in the previous 3 years, aged 31-50 years old  E: NR | 40.2  (5.4) | 62 | 77.5  (16.7) | 174.6  (8.9) | 25.2  (3.7) | N/A | N/A |
| Ritvanen (2007);  Finland; Laboratory; RCT | Pain  (Traditional bone setting) | B: 35  A: 33 | I: Between 20-60 years old, had LBP that restricted functioning and had LBP present on at least half of the days in a 12-month period in a single episode or in multiple episodes  E: Referred pain distal to knee, severe neurologic, metabolic, or cardiovascular diseases, back surgery, mental diseases, major structural abnormality (e.g., kyphoscoliosis), pensionable disease, pregnancy | M:  40.8  (5.5)  F:  40.6  (3.99) | 45.4 | M:  77  (11)  F:  70  (17) | M:  176  (3)  F:  165  (6) | M:  24.8  (5.6)  F:  25.6  (5.7) | 7  (7) | VAS (/100):  B – 40  (4)  ODI (%):  B – 18  (2)  Depression Score (/21):  B – 3.73  (2.7)  Finger-floor Distance (cm):  B – 5.8  (1.8)  Lateral Bending Right (cm):  B – 16.1  (0.7)  Lateral Bending Left (cm):  B – 16.1  (0.6) |
|  | Pain  (Physical therapy) | B: 35  A: 28 | I: Same as Traditional bone setting group  E: Same as Traditional bone setting group | M:  41  (5.9)  F:  42  (6) | 43 | M:  80  (9)  F:  69  (10) | M:  177  (6)  F:  167  (5) | M:  25.5  (2.8)  F:  24.7  (3.6) | 11  (8) | VAS (/100):  B – 41  (4)  ODI (%):  B – 21  (2)  Depression Score (/21):  B – 4.03  (2.09)  Finger-floor Distance (cm):  B – 5.9  (1.9)  Lateral Bending Right (cm):  B – 16.5  (0.5)  Lateral Bending Left (cm):  B – 16.3  (0.8) |
| Salamat (2017); Iran;  Laboratory;  RCT | Pain  (Stabilization exercises) | B: 16  A: 12 | I: Non-specific chronic low back pain duration > 3 months at the lower lumbar segments (L4-5 or L5-S1), aggravation of symptoms in movements or postures related to extension direction, excess of lumbar lordosis at symptomatic levels, Tampa Scale of Kinesiophobia questionnaire scores < 41, Oswestry Disability Index >13%  E: Specific back pain such as vertebral fracture and spondylolisthesis, previous low back pain with leg pain, previous motor control exercise therapy and pregnancy. People with evidence of distress (based on Start Back questionnaire, score >4 or considered high risk based on Start Back score) | 35.83  (9.31) | NR | 64.33  (10.65) | 1.63  (5.81)* | 23.92  (4.25) | NR | NPRS (/10):  B – 5.16  (1.74)  ODI:  B – 22.16  (8.8)  TSK:  B – 36.7  (5.5) |
|  | Pain  (Movement control exercises) | B: 16  A: 12 | I: Same as Stabilization exercises group  E: Same as Stabilization exercises group | 36.09  (9.6) | NR | 64  (9.41) | 1.64  (5.94) | 23.75  (2.35) | NR | NPRS (/10):  B – 5.9  (1.92)  ODI:  B – 24.63  (10.85)  TSK:  B – 35.7  (8.34) |
| Shamsi (2022b); Iran;  Physio Clinic;  RCT | Pain  (Static stretching) | 15 | I: Low back pain (pain of the posterior aspect of the body from the lower margin of the twelfth ribs to lower gluteal folds) > 3 months, pain intensity between 3 and 6 on VAS, obvious hamstring shortness determined by straight leg raise, 18 to 60 years  E: Pathology or anomaly of lower limbs (e.g., neuropathic pain, malignancy, inflammatory diseases, severe osteoporosis, arthritis, bone diseases) | 37.67  (8.96) | 33 | 76.57  (13.05) | 171.93  (13.21) | 25.90  (3.15) | NR | VAS:  B – 52.62  95%CI  [42.74,62.51]  ODI:  B – 41.00  95%CI  [32.43,49.57] |
|  | Pain  (Strength) | 15 | I: Same as Static stretching group  E: Same as Static stretching group | 37.07  (13.39) | 27 | 81.54  (16.59) | 172.64  (10.14) | 26.82  (4.21) | NR | VAS:  B – 61.15  95%CI  [53.92,68.40]  ODI:  B – 43.67  95%CI  [35.45,51.88] |
|  | Pain  (No intervention control) | 15 | I: Same as Static stretching group  E: Same as Static stretching group | 39.12  (11.61) | 33 | 80.91  (14.1) | 172.31  (10.14) | 27.47  (3.17) | NR | VAS:  B – 61.13  95%CI  [50.37,71.89]  ODI:  B – 35.50  95%CI  [29.49,41.51] |
| Ting (2017); USA;  Laboratory;  Single-arm clinical trial | Pain | B: 82  A: 76  (B)  A: 69  (2wk)  A: 67  (6wk) | I: Adults aged 21-65 years old, meeting the Quebec Task Force for Spinal Disorders (QTF) classification of 1, 2 or 3, reported LBP duration for more than 12 weeks, scored ≥6 on the Roland Morris Disability Questionnaire during a voice phone screen conducted by study personnel before the first baseline visit, a patient-reported average pain over the past 24hr ≥ 2 on an 11 point numerical rating scale at phone screen and baseline visits 1 and 2 (BL1 and BL2)  E: Additional diagnostic tests or urgent/emergent procedures needed beyond study exam procedures, BMI ≥ 40, BDI II score ≥ 29, compliance concerns, co-morbidity requiring coincident clinical management, inability to read or verbally comprehend English, inflammatory or destructive tissue changes to the spine, join replacement history, moving from the area within 8 weeks, neuromuscular disease, no indication for spinal manipulation (SM) at L1-L5 or sacroiliac joints, open or pending litigation for LBP or seeking/receiving disability compensation, pacemaker/defibrillator, peripheral artery disease, pregnancy, QTF classification 4-11, received SM within past 4 weeks, safety, suspicion of drug or alcohol abuse, uncontrolled hypertension | 44.9  (10.6) | 48 | NR | NR | 30.6  (5.3) | 12w-6mo: 7  6mo-1y:  6  >1y:  69 | QTF:  B –  Class 1: 55  Class 2: 19  Class 3: 8 |
| Watson (1997); United Kingdom;  Laboratory;  Cross-sectional | Pain | 36 | I: Patients attending the Manchester and Salford Back Pain Centre for the Interdisciplinary Pain Management Program, between 18-65 years old, chronic low back pain (CLBP) primary presenting condition, duration of pain of at least 6 mo, not undergoing any current treatment than current analgesia, willing to participate in a pain management program  E: Evidence of a major structural abnormality (e.g., kyphoscoliosis); inflammatory, systemic or neoplastic disease, major psychiatric illness, pregnancy, other medical conditions likely to interfere with an active rehabilitation program | 43.7  (9.3) | 58 | NR | NR | NR | 4.6  (4.2) | VAS:  B – 49.6  (22.6)  FABQ:  B – 15.9  (4.9)  ODI:  B – 51.1  (13.9)  PSEQ:  B – 25.1  (11.8) |

A = Analysis, B = Baseline, E = Exclusion Criteria, F = Females, FABQ = Fear Avoidance Beliefs Questionnaire, FABQ-PA = Fear Avoidance Beliefs Questionnaire for physical activity, FABQ-W = Fear Avoidance Beliefs Questionnaire for Work, F/U = Follow up, I = Inclusion Criteria, IQR = Interquartile Range, NPRS = Numeric Pain Rating Scale, NR = Not Reported, VAS = Visual Analoge Scale, M = Males, mODI = Modified Oswestry Disability Questionnaire, N/A = Not Applicable, NDI = Neck Disability Index, ODI = Oswestry Disability Index, PSEQ = Pain Self-Efficacy Questionnaire, QTF = Quebec Task Force Classification, RMDQ = Roland Morris Disability Questionnaire, RAPA-A = Rapid Assessment of Physical Activity – Aerobic, RAPA-SF = Rapid Assessment of Physical Activity - Strength and Flexibility, SEES = Self-efficacy for Exercise Scale, SF-12 = 12-item Short Form Survey, SF-MPQ = Short Form McGill Pain Questionnaire, TSK = Tampa Scale of Kinesiophobia, 95%CI = 95 Percent Confidence Interval.
