# Supplementary figures and images for: A systematic review and meta analysis of measurement properties for the flexion relaxation ratio in people with and without non specific spine pain
Source: Sci Rep. 2024 Feb 8;14:3260. doi: 10.1038/s41598-024-52900-z (PMC10853169; doi:10.1038/s41598-024-52900-z)

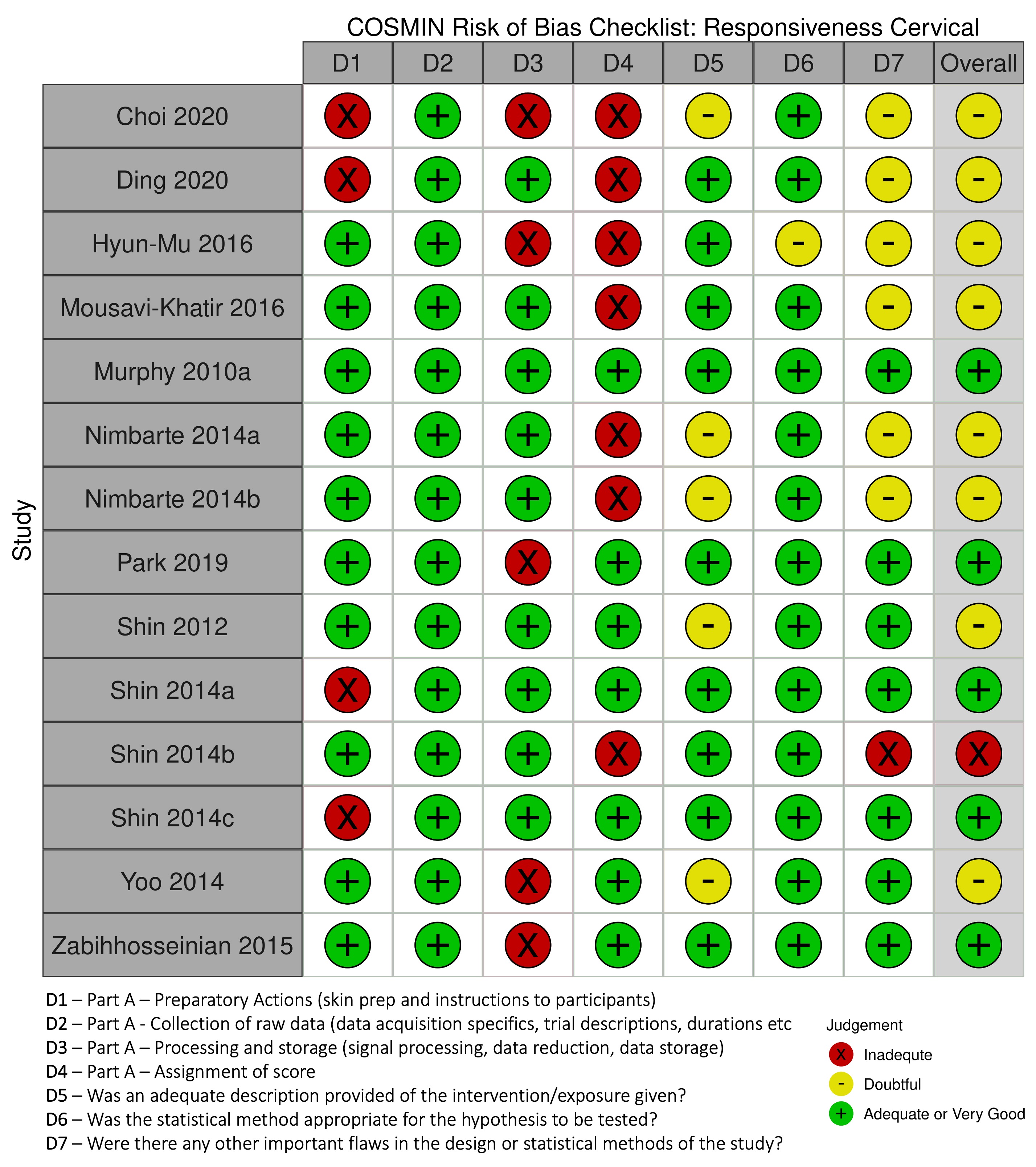

Supplement: Supplementary file 8 — Supplementary Figure. [file 41598_2024_52900_MOESM8_ESM.jpg]

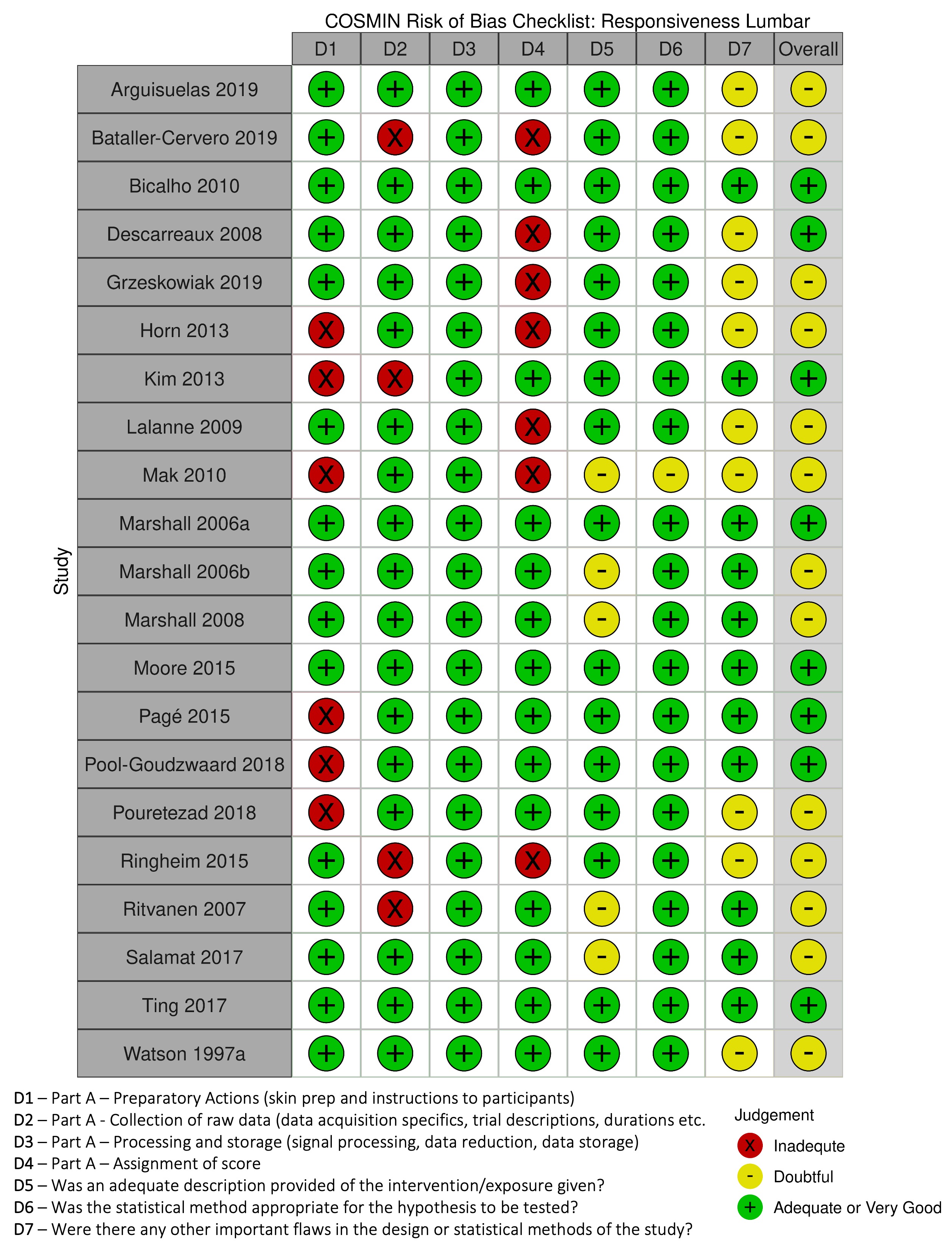

Supplement: Supplementary file 9 — Supplementary Figure. [file 41598_2024_52900_MOESM9_ESM.jpg]
