# Supplementary material for: A systematic review and meta analysis of measurement properties for the flexion relaxation ratio in people with and without non specific spine pain
Source: Sci Rep. 2024 Feb 8;14:3260. doi: 10.1038/s41598-024-52900-z (PMC10853169; doi:10.1038/s41598-024-52900-z)
Supplement: Supplementary file 10 — Supplementary Table 3f. [file 41598_2024_52900_MOESM10_ESM.docx]

Supplementary Table 3f – Summary of exposure types and specifics, and risk of bias and good measurement property results for the included studies of responsiveness for the cervical flexion relaxation ratio.

| Exposure Type | Specifics | Author | ROB | Good Measurement Property |
| --- | --- | --- | --- | --- |
|  | Prolonged Sitting | Choi et al. 2020 | Doubtful | - |
|  | End Range Cervical Flexion (10 minute) | Mousavi-Khatir et al. 2016 | Doubtful | + |
|  | Smartphone posture desk vs lap | Shin and Kim 2014c | Adequate | - |
|  | Overhead work | Shin et al 2012 | Doubtful | + |
|  | VDT Entry (30 minutes) | Shin et al 2014b | Inadequate | - |
|  | Below Knee Assembly Work | Shin et al., 2014a | Very Good | + |
|  | Assembly Work | Yoo et al., 2014 | Doubtful | + |
| Therapeutic Interventions | Surgery for Knee Flexion Contracture | Ding et al., 2016 | Doubtful | + |
|  | Exercise | Hyun-Mu et al., 2016 | Doubtful | + |
|  | Exercise (Group 2) | Murphy et al., 2010a | Very Good | - |
|  | SMT (Group 1) |  |  |  |
|  | Static Stretching | Park et al., 2019 | Very Good | + |
|  | Dynamic Stretching |  |  |  |
| Fatigue Protocols |  | Nimbarte 2014a | Doubtful | + |
|  |  | Nimbarte 2014b | Doubtful | + |
|  |  | Zabihhosseinian et al., 2015 | Adequate | + |
