# Supplementary material for: A systematic review and meta analysis of measurement properties for the flexion relaxation ratio in people with and without non specific spine pain
Source: Sci Rep. 2024 Feb 8;14:3260. doi: 10.1038/s41598-024-52900-z (PMC10853169; doi:10.1038/s41598-024-52900-z)
Supplement: Supplementary file 11 — Supplementary Table 3g. [file 41598_2024_52900_MOESM11_ESM.docx]

Supplementary Table 3g – Summary of exposure types and specifics, and risk of bias and good measurement property results for the included studies of responsiveness for the lumbar flexion relaxation ratio.

| Exposure Type | Specifics | Author | ROB | Good Measurement Property |
| --- | --- | --- | --- | --- |
| Prolonged Postures | Prolonged Standing (15 minute) | Ringheim et al., 2015 | Doubtful | + |
|  | Below Knee Assembly Work | Shin et al., 2014a | Very Good | + |
| Therapeutic Interventions | Myofascial Release (2 sessions, 2 wks apart) | Arguisuelas et al., 2019 | Doubtful | - |
|  | Lumbar Support (2 month) | Bataller-Cervero et al., 2019 | Doubtful | - |
|  | SMT | Bicalho et al., 2010 | Very Good | + |
|  | Kinesiotape | Greześkowiak et al., 2019 | Doubtful | - |
|  | Physical Therapy (40 min, 4x/wk) | Kim et al., 2013b | Very Good | + |
|  | Lumbar Stabilization Exercises (40 min, 4x/week) |  |  |  |
|  | SMT | Lalanne et al., 2009 | Doubtful | - |
|  | Rehabilitation (5 sessions/wk, 12 weeks) | Mak et al., 2010 | Doubtful | + |
|  | Exercise Intervention | Marshall and Murphy 2006a | Very Good | + |
|  | Exercise Intervention | Marshall and Murphy 2006b | Doubtful | + |
|  | SMT compared to control and Combo w/Swiss Ball | Marshall et al., 2008 | Doubtful | + |
|  | Stretching (5-week program) | Moore et al., 2015 | Very Good | - |
|  | Flexion/Extension Exercise (Biofeedback) | Pagé et al., 2015 | Very Good | + |
|  | Traditional Bone Setting (5x @ 2-week intervals) | Ritvanen et al., 2007 | Doubtful | + |
|  | Physical Therapy (5x @ 2-week intervals) |  |  |  |
|  | Stabilization Exercises (8x over 4 wks) | Salamat et al., 2017 | Doubtful | + |
|  | Movement Control Exercises (8x over 4 wks) |  |  |  |
|  | Stretching (3x/week 4 weeks) | Shamsi et al., 2022 | Very Good | - |
|  | Strengthening (3x/week over 4 weeks) |  |  |  |
|  | Control |  |  |  |
|  | SMT | Ting et al, 2017 | Adequate | - |
| Fatigue Protocols | Sustained Contraction to Fatigue | Descarreaux et al., 2008 | Very Good | - |
|  | Exercise to Fatigue | Horn et al., 2013 | Doubtful | - |
| Behavioural/ Cognitive | Pick up wallet after watching videos of movement performed with different emotions | Pool-Goudzwaard et al., 2018 | Adequate | + |
|  | Bending with Cognitive Task | Pouretezad et al., 2018 | Doubtful | + |
|  | 15-day Cognitive Behavioural Therapy Program | Watson et al., 1997 | Doubtful | + |
